# Efficacy of three anti-malarial regimens for uncomplicated *Plasmodium falciparum* malaria in Cambodia, 2009–2011: a randomized controlled trial and brief review

**DOI:** 10.1186/s12936-022-04279-3

**Published:** 2022-09-07

**Authors:** Dysoley Lek, Agus Rachmat, Dustin Harrison, Geoffrey Chin, Suwanna Chaoratanakawee, David Saunders, Didier Menard, William O. Rogers

**Affiliations:** 1 National Centre for Parasitology, Entomology and Malaria Control, Phnom Penh, Cambodia; 2 U.S. Naval Medical Research Unit 2, Phnom Penh, Cambodia; 3 Uniformed Services University of the Health Sciences, Bethesda, MD USA; 4 Faculty of Public Health, Mahidol University, Bangkok, Thailand; 5 Institut Pasteur du Cambodge, Phnom Penh, Cambodia

**Keywords:** Uncomplicated falciparum malaria, Cambodia, Antimalarial resistance, Randomized clinical trial, Therapeutic efficacy

## Abstract

**Background:**

Anti-malarial resistance remains an important public health challenge in Cambodia. The effectiveness of three therapies for uncomplicated falciparum malaria was evaluated in Oddar Meanchey province in Northern Cambodia from 2009 to 2011.

**Methods:**

In this randomized, open-label, parallel group-controlled trial, 211 subjects at least 5 years old with uncomplicated falciparum malaria were treated with 3 days of directly observed therapy: 63 received artesunate–mefloquine (AS/MQ), 77 received dihydroartemisinin–piperaquine (DHA/PPQ), and 71 received atovaquone–proguanil (ATQ/PG). The subjects were followed for 42 days or until recurrent parasitaemia. Genotyping of *msp1*, *msp2*, and *glurp* among individual parasite isolates distinguished recrudescence from reinfection. *Pfmdr1* copy number was measured by real-time PCR and half-maximal parasite inhibitory concentrations (IC_50_) were measured in vitro by 48-h isotopic hypoxanthine incorporation assay.

**Results:**

The per-protocol PCR-adjusted efficacy (95% confidence interval) at 42 days was 80.6% (70.8–90.5%) for AS/MQ, 97.2% (93.3–100%) for DHA/PPQ, and 92.9% (86.1–99.6%) for ATQ/PG. On day 3, 57.9% remained parasitaemic in the AS/MQ and DHA/PPQ arms. At baseline, 46.9% had microscopic *Plasmodium falciparum* gametocytaemia. Both recurrences in the DHA/PPQ arm lost *Pfmdr1* copy number amplification at recrudescence. All four recurrences in the ATQ/PG arm were wild-type for cytochrome bc_1_. One subject withdrew from the ATQ/PG arm due to drug allergy.

**Conclusions:**

This study was conducted at the epicentre of substantial multi-drug resistance that emerged soon thereafter. Occurring early in the national transition from AS/MQ to DHA/PPQ, both DHA/PPQ and ATQ/PG had acceptable efficacy against uncomplicated falciparum malaria. However, efficacy of AS/MQ was only 80% with apparent mefloquine resistance based on elevated *Pfmdr1* copy number and IC_50_. By 2009, there was already significant evidence of artemisinin resistance not previously reported at the Northern Cambodia–Thai border. This study suggests the basis for early development of significant DHA/PPQ failures within 3 years of introduction. Artemisinin resistance likely occurred on the Northern border concurrently with that reported along the Western border in Pailin.

*Trial registration* This legacy trial was conducted prior to International Committee of Medical Journal Editors’ requirements for preregistration on ClinicalTrials.gov. The full protocol has been provided.

**Supplementary Information:**

The online version contains supplementary material available at 10.1186/s12936-022-04279-3.

## Background

The spread of drug-resistant *Plasmodium falciparum* has complicated efforts to control malaria. This may lead to unnecessary mortality if ineffective drugs remain the standard of care after drug-resistant strains become established [1, 2]. In Southeast Asia, resistance is common to multiple anti-malarial drugs including chloroquine (CQ), sulfadoxine–pyrimethamine, quinine (QN) and mefloquine (MQ) [3]. Given the worsening situation, countries in the region adopted short-course artemisinin-based combination therapy (ACT) as first-line treatment for uncomplicated *P. falciparum* malaria. In Cambodia, current Ministry of Health guidelines have returned to the use of artesunate–mefloquine (AS/MQ) as first-line therapy for uncomplicated *P. falciparum* malaria [4]. This followed several years where dihydroartemisin–piperaquine (DHA/PPQ) had replaced AS/MQ due to widespread clinical resistance to the latter, and evidence of inverse resistance patterns to the two artemisinin-based combinations [5].

Mefloquine was first introduced along the Thai-Cambodian border in 1983 [6]. In 1994, AS/MQ became the first ACT used along the Thai–Myanmar border due to increasing MQ resistance [7]. By 1995, AS/MQ became first-line therapy for uncomplicated falciparum malaria in Thailand, with Cambodia following in 2000 [6]. In 2003, significant clinical AS/MQ failures were documented at Trat, Thailand [8], then in the neighbouring Western Cambodian province of Pailin in 2002 and 2004 [9]. In Chumkiri, a Southern Cambodian province far from the Thai border, an unacceptably low rate of adequate clinical and parasitological response (ACPR) was observed for AS/MQ in 2006–2007 [10]. Due to rising AS/MQ failure rates, Western Cambodia adopted DHA/PPQ in 2008, with the rest of the country following in 2012 [11]. Shortly after significant DHA/PPQ failures were reported in Oddar Meanchey province [12], Cambodia transitioned back to AS/MQ in regions of significant DHA/PPQ failures with low *Pfmdr1* copy numbers [13].

**Fig. 1:**
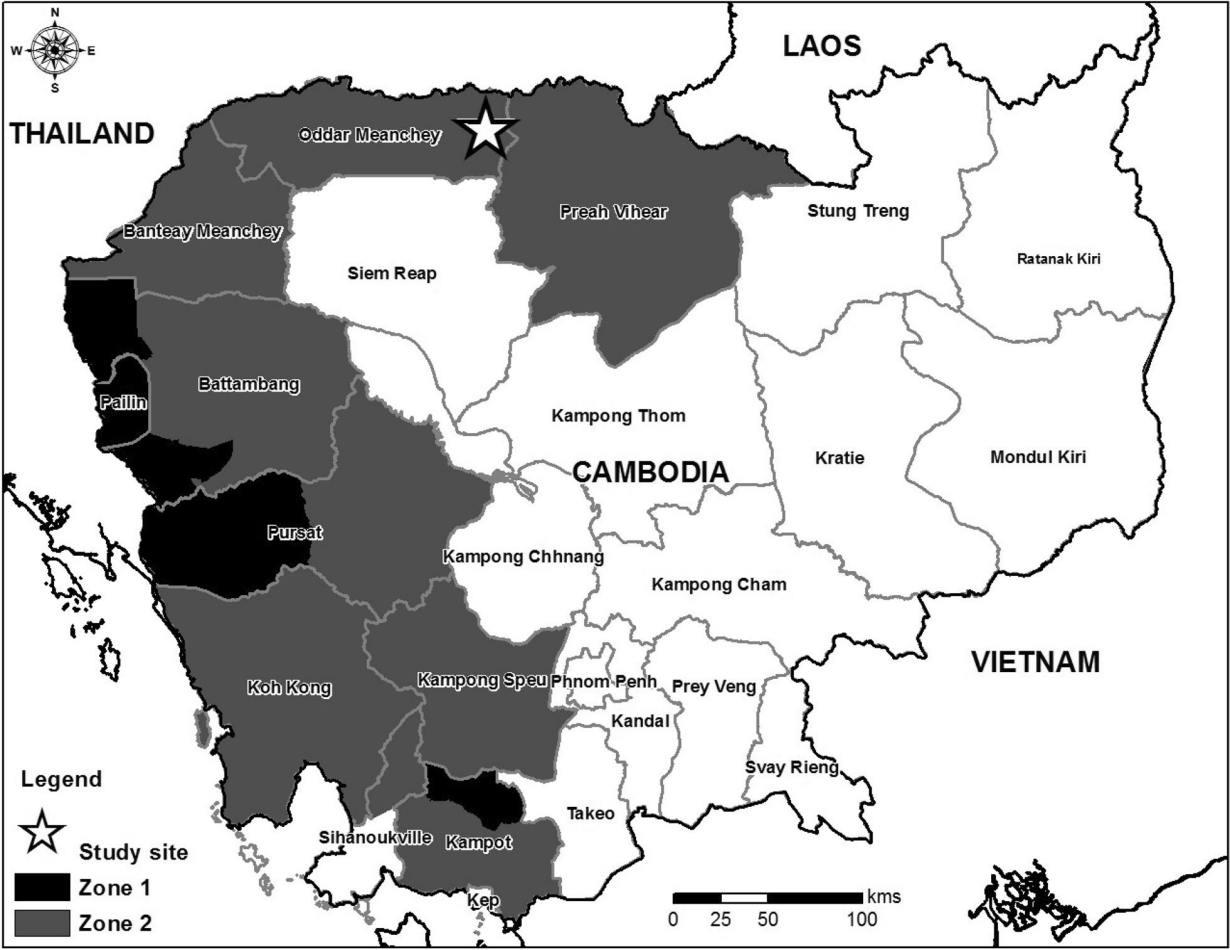
Map of Cambodia with Phase 1 and 2 Zones. Site of the 2009 study in relation to previously established malaria containment Zones 1 and 2. Zone 1 was considered the highest risk and had already switched to the use of DHA/PPQ. In addition, there were monitored mass screening and treatment activities using ATQ/PG for PCR-identified subclinical *P. falciparum* malaria cases

The Cambodian Ministry of Health took significant steps to contain the possible spread of *P. falciparum* resistant to AS/MQ. In collaboration with the World Health Organization (WHO) and the Gates Foundation, the Ministry of Health designed a programme to confirm the presence of artemisinin resistance, and to contain its spread. The plan defined Phase 1 and Phase 2 containment and elimination zones (Fig. 1), including areas where significant failure of AS/MQ was documented (Phase 1). Phase 2 areas adjacent to Phase 1 were thought to be at higher risk.

In the Phase 1 containment area, AS/MQ was replaced by DHA/PPQ as first-line therapy for uncomplicated *P. falciparum* malaria. The efficacy and tolerability of DHA/PPQ were previously reviewed in 2007 [5] with 14 studies involving 2,636 patients with uncomplicated falciparum malaria having 28 day cure rates of 97–98%. Therapy was well tolerated with common adverse events of nausea, vomiting, anorexia, headache, dizziness, diarrhoea, and abdominal pain occurring in 1–10% of subjects—notably also common symptoms of malaria. There were no serious adverse events reported.

The success of the containment programme was dependent on the extent to which AS/MQ resistance had already spread beyond the Phase 1 containment zone, and on the continuing efficacy of DHA/PPQ and ATQ/PG in treating uncomplicated *P. falciparum* in the Phase 2 containment zone. In 2008, the WHO identified ATQ/PG as the only realistic non-artemisinin-based anti-malarial, which had never been used at the time in Cambodia [14]. At the time of the study, the Ministry of Health conducted a large-scale screening and treatment campaign in the Phase 1 zones using PCR for diagnosis of inapparent infections and atovaquone–proguanil (ATQ/PG) therapy. In previous studies, NAMRU-2 identified sites at risk for AS/MQ resistance by screening approximately 700 parasite samples from cases of uncomplicated falciparum malaria from 5 provinces within and outside the containment zones for the prevalence of amplified *Pfmdr1* gene. Amplifications at this locus are strongly associated with in vivo resistance to mefloquine [15]. The highest average *Pfmdr1* copy number (~ 2.5) was found, unsurprisingly, at Chumkiri, the site in southern Cambodia where NAMRU-2 previously identified significant resistance to AS/MQ [10]. Sites in eastern Cambodia, outside both containment zones, had average *Pfmdr1* copy numbers < 1.5, but a site in containment zone 2, Trapeang Prasat, Oddar Meanchey province, had an elevated mean *Pfmdr1* copy number (~ 2), suggesting a risk of AS/MQ resistance (unpublished data). This study was undertaken in 2009 to assess the in vivo efficacy of AS/MQ, DHA/PPQ, and ATQ/PG at this site, following guidelines recommended by the WHO and the Worldwide Antimalarial Resistance Network (WWARN) [16, 17].

## Methods

### Design

This study was an open-label, parallel group, randomized controlled trial evaluating the efficacy of 3 standard anti-malarial regimens for the treatment of uncomplicated falciparum malaria. The purpose was to evaluate therapeutic efficacy of the first-line treatment (AS/MQ) at the time of the study in addition to its potential replacements (DHA/PPQ and ATQ/PG) to guide decision-making and policy, as described in WHO guidelines [17]. Subjects who agreed to participate in the study were randomized to one of three arms—AS/MQ, DHA/PPQ, or ATQ/PG. Subjects were treated with directly observed therapy for 3 days and followed for up to 42 days or until evidence of recurrent parasitaemia. The protocol has been provided as Additional file 1.

### Study location

The study was conducted at the Trapeang Prasat Health Centre in Oddar Meanchey Province, Cambodia (Fig. 1). This site is in a rural, agricultural area about 8 h by road from Phnom Penh. Oddar Meanchey province was in the Phase 2 containment zone and the health centre participated in a surveillance programme to measure the prevalence of mutations in *P. falciparum* drug resistance markers.

### Study enrolment

Subjects were recruited from areas around the study site and included if they met the following criteria: (1) age ≥ 5 years, (2) blood-stage *P. falciparum* parasitaemia > 1000 but < 100,000 asexual parasites per µL, (3) axillary temperature > 37.5 °C or rectal or tympanic temperature > 38.0 °C, or history of documented fever within past 24 h, (4) negative urine pregnancy test, (5) ability to swallow oral medication, (6) availability for follow-up over 42 days, and (7) freely provided informed consent. Exclusion criteria were: (1) age < 5 years, (2) mixed infection with *Plasmodium vivax*), (3) positive urine human chorionic gonadotrophin test for pregnancy, (4) history of epilepsy or psychiatric illness, (5) meeting WHO criteria for severe malaria [2], (6) serious co-morbid conditions requiring hospitalization (including, but not limited to severe renal or liver disease, uncontrolled diabetes, systemic bacterial infections), (7) on-going antibiotic therapy, (8) history of hypersensitivity to any of study drug, (9) plans to leave area during next 42 days or to be unavailable for scheduled follow-up, (10) nursing mother, and (11) any other condition which, in the judgment of the study physician would make participation in the study unsafe for the potential volunteer. Eligible, consenting subjects were enrolled; ineligible persons were referred for routine therapy in the clinic.

### Randomization and dosing

A statistician, otherwise unconnected with the study, randomly distributed cards indicating one of the three regimens into 300 sequentially numbered envelopes, which were sealed and signed. When a subject provided informed consent and enrolled in the study, the envelope corresponding to the subject’s study number was opened by a member of the study team. Medications were provided by the Cambodian Ministry of Health and were dosed according to National Guidelines as described in the protocol (Additional file 1).

Artesunate–mefloquine: One tablet of AS contained 50 mg and one tablet of MQ contained 250 mg; dosage was based on weight (12 mg/kg of AS and 25 mg/kg of MQ) and up to a maximum of 600 mg AS and 1250 mg MQ total over 3 days.

Dihydroartemisinin–piperaquine: One tablet contained 40 mg of DHA and 320 mg of PPQ; dosage was based on weight and up to a maximum of 360 mg DHA and 2880 mg PPQ over 3 days.

Atovaquone–proguanil: One tablet contained 250 mg of ATQ and 100 mg of PG; dosage was based on weight and up to a maximum of 3000 mg ATQ and 1200 mg PG.

### Study procedures

Microscopists and laboratory technicians were blinded to the treatments each subject received. Patients were observed in the clinic for one hour after each dose. Before initiating therapy, up to 5 mL venous blood was drawn for in vitro drug resistance assays, resistance markers, and genotyping studies. Subjects were asked to re-visit the study unit on days 1, 2, 3, 7, 14, 18, 21, 28, 35 and 42. Subjects were also instructed to return on any other day that they felt ill in order to monitor clinical recovery/recurrence of malaria symptoms. In the event that a subject failed to re-visit the study unit on schedule, a member of the study team visited the subject. Treatment failures (persistent or recurrent parasitaemia) were treated with oral quinine sulphate 10 mg salt/kg three times daily and tetracycline 1 g daily for 7 days.

### Malaria diagnosis

Thick and thin blood smears were stained with 10% Giemsa for 10 min and examined by a certified microscopist using 1000× oil immersion light microscopy. At least 200 ocular fields were examined and the number of asexual and sexual forms per 200 white blood cells (WBC) in the thick smear were recorded separately. This was converted to parasites/µL for analysis using a conversion multiple of 40 (assuming 8000 WBC/µL).

#### In vitro resistance assays

The in vitro drug sensitivity of the *P. falciparum* isolates was assessed by use of a classical isotopic 48-h test [18, 19]. Stock solutions ofanti-malarial drugs (Sigma-Aldrich, Singapore) were prepared in methanol and further twofold serial dilutions in distilled water (Biosedra, France). Two wells of a Falcon 96-well, flat-bottom plate (ATGC, France) were coated with each drug concentration, dried and stored at 4 °C until use. In vitro testing used the 3D7 and Dd2 reference strains of *P. falciparum* with known drug sensitivities. Blood samples with a parasitaemia of at least 6400 asexual parasites/µL were washed three times with RPMI 1640 medium (GibcoTM, Invitrogen Corporation, France) by centrifugation (800×*g*, 10 min, 4 °C) and tested directly without culture adaptation. Infected erythrocytes were suspended (1.5% haematocrit, 0.1–1% parasitaemia) in complete RPMI medium supplemented with 10% decomplemented human AB+ serum (Biomedia, France) buffered with 25 mM HEPES and 11 mM d-(+)-glucose 25 mM NaHCO_3_, containing [G-^3^H] hypoxanthine (0.5 µCi/well; Amersham Biosciences, France).

The mixture was distributed (200 µL per well) into 96-well test plates pre-coated with anti-malarial drugs. Each plate included two drug-free control wells and one control well without parasites. Plates were incubated for 48 h at 37 °C in a 5% CO_2_ atmosphere and then the cells were lysed by freeze–thawing. Following collection on glass-fibre filter paper using a cell harvester, the amount of [G-^3^H]hypoxanthine incorporated into the parasites’ nucleoprotein was determined using a Wallac MicroBeta Trilux counter (Perkin Elmer, France). A log probit approximation was used to determine the 50% inhibitory concentration (IC_50_), defined as the concentration at which 50% of [G-^3^H]hypoxanthine incorporation was inhibited compared to drug-free control wells. The in vitro resistance assay required a starting parasitaemia of at least 0.1% and was not performed on samples with a starting parasitaemia < 6400 parasites/µL (160 parasites/200 WBC). The IC_50_ thresholds for in vivo resistance to CQ, MQ, and QN have been described previously for radioisotope assays using culture adapted *P. falciparum* isolates: IC_50_ to CQ > 45.5 ng/mL (85 nM), IC_50_ to MQ > 10 ng/mL (24 nM), and IC_50_ to QN > 275 ng/mL (351 nM) [20, 21].

### Parasite genotyping studies

Fifty µL of venous blood was spotted on #1 Whatman Filter Paper and stored individually in zip-lock plastic bags with desiccant. Dried blood blots were cut into small pieces, placed in 1.5 ml microcentrifuge tube and lysed in 1 ml of sterile water for 10 min at room temperature. During this step, the tubes were vortexed every 1–2 min and then centrifuged at 4500×*g* for 5 min. The supernatant was decanted, and DNA precipitate was resuspended in 10 volumes of 5% chelex-100 (Biorad Laboratories Inc. Hercules, CA), and incubated for 20 min at 56 °C with a brief vortex before and after incubation. Tubes were then placed in a 100 °C heating block for 8 min and vortexed briefly after incubation. After a final centrifugation at 4500×*g* for 2 min to precipitate chelex-bound organic material, DNA-containing supernatants were removed by pipette for PCR amplification. Blood spots of about 2 cm diameters yielded 50 to 100 ng total DNA.

Genotyping to distinguish new infections from recrudescences was conducted by amplification of the merozoite surface proteins 1 and 2 (*msp1* and *msp2*) and glutamine-rich protein (*glurp*) markers on paired samples obtained from participants on day 0 and the day of failure. Primers designed to amplifiy three allelic families from block two of *msp1* (K1, MAD20, R033), two allelic families from *msp2* (FC27 and 3D7), and the polymorphic region of *glurp* were used in PCR amplification and analysis as previously described [22]. The paired samples’ PCR products were loaded adjacent to each other. Gels were stained with ethidium bromide and visualized under UV illumination. Band sizes of PCR products across the three markers were visualized under UV illumination. Band sizes of PCR products across the three markers were measured visually and compared for paired day 0 and day of failure samples. In accordance with WHO recommendations, a recurrent infection was classified as recrudescent infection (treatment failure) if there was at least one matching band in any allelic family for all three markers [23]. If there were no shared alleles for at least one marker, a recurrent infection was classified as a reinfection. If the amplification failed for a marker, the marker was not used for reinfection and recrudescence determination, but the aforementioned classification criteria were applied for the markers that were amplified.

Analysis of amplification of the *Pfmdr1* gene was performed as described elsewhere [24, 25]. Multiplex PCR was used to amplify products from both the *Pfmdr1* and *β-tubulin* genes in a single tube. PCR was performed in a total volume of 25 µL containing Rotor-gene Probe PCR Kit (containing PCR buffer and HotStarTaq Plus DNA Polymerase), 3.0 mM MgCl_2_, 300 µM of each deoxynucleoside triphosphate, 300 nM each *Pfmdr1* primer (forward primer: 5′-ttaagttttactctaaaagaagggaaaacatat-3′; reverse primer: 5′-tctccttcggttggatcataaag-3′) with 150 nM of *Pfmdr1* probe labelled with 5′ Fam and 3′ Tamra (5′-Fam-catttgtgggagaatcaggttgtgggaaat-Tamra-3′) [24], 100 nM of each *β-tubulin* primer (forward primer: 5′-tgatgtgcgcaagtgatcc-3′; reverse primer: 5′-tcctttgtggacattcttcctc-3′) with 100 nM of *β-tubulin* probe labelled with 5′ Vic and 3′ Tamra (5′-Vic-tagcacatgccgttaaatatcttccatgtct-Tamra-3′ [25]. Each assay was performed in duplicate. Reactions were carried out in a Rotorgene real-time PCR system using the following cycling conditions: 95 °C for 3 min, 50 cycles of 95 °C for 15 s, and 60 °C for 1 min. Results were accepted as valid if the copy number for control DNA samples were 0.8–1.2 for 3D7 and 2.8–3.2 for W2Mef, and if the difference between duplicate copy numbers was < 50% of the average. The efficiency $E$, was calculated from the slope of a standard curve made from known dilutions of a reference DNA $(E = 10 - 1/slope)$. The copy number was calculated as: $Copy\# = \left( {Ebt*Ctbt} \right)/\left( {Emdr*Ctmdr} \right)$ where $Ebt$ and $Emdr$ are the efficiencies for *β-tubulin* and *Pfmdr1*, respectively, and $Ctbt$ and $Ctmdr$ are the corresponding number of cycles to reach threshold.

*Plasmodium falciparum* isolates were screened for the cytochrome b Y268 mutations that have been associated with atovaquone resistance. Briefly, outer PCR products (1385 base pairs) were used as templates for two second-round [26]. For both nested PCRs (NsiI and SspI), amplification took place in the following reaction mixture: 2.5 mL of buffer, 1.5 mM MgCl_2_, 0.2 mM each deoxynucleoside triphosphate, 0.5 mM each primer (NsiI forward primer: 5′-ggtttacttggaacagtttttaacaatg-3′, NsiI reverse primer: 5′-ggtttacttggaacagtttttaacaatg-3′, SspI forward primer: 5′-acagaataatctctagcacc-3′, SspI reverse primer: 5′-acctgaatggtactttctacaatat-3′), 2 units of FirePol Taq polymerase (Solid Biodyne, Estonia), and 2 mL of PCR products. Nested PCRs were performed under the following conditions: heated at 94 °C for 5 min, followed by 30 cycles of heating (94 °C for 30 s, 45 °C for nested PCR NsiI or 55 °C for nested PCR SspI for 90 s, and 72 °C for 2 min), and heated for a final extension period at 72 °C for 10 min. Five mL of nested PCR products, adjusted to 500 ng/mL, were respectively mixed with 0.2 mL of NsiI and 2.5 mL of buffer 3 (for nested PCR NsiI) or with 0.2 mL of SspI and 2.5 mL of buffer 2 (for nested PCR SspI) according to the manufacturer’s instructions (New England BiolabsH, France), incubated for 4 h at 37 °C, and inactivated at 65 °C (for nested PCR SspI) or 80 °C (for nested PCR NsiI) for 20 min. Bands were detected by standard 2% agarose gel electrophoresis and ethidium bromide staining. Polymorphisms at codon 268 were assessed according to the number and size of the bands: Y268Y (tat) − NsiI restriction fragment length polymorphism (RFLP) (2 bands: 359 + 25 base pairs) and SspI (2 bands: 151 + 23 base pairs); Y268N (aat) − NsiI RFLP (2 bands: 359 + 25 base pairs) and SspI (1 band: 174 base pairs); Y268C (tgt) or Y268S (tct) − NsiI RFLP (1 band: 384 base pairs) and Sspl (2 bands: 151 + 23 base pairs). Mutations at codon 268 were secondarily confirmed by sequencing. Briefly, nested PCR products were sent to Macrogen (Seoul, South Korea). Electrophoregrams were visualized and analysed with CEQ2000 software v2.0 (Beckman Coulter, France). Amino acid sequences were compared with the sequence of 3D7 reference strain (PF3D7_MIT02300).

### Sample size

Based on the primary hypothesis that the upper bound of the 95% confidence interval for the proportion of ACPR in subjects treated with AS/MQ is < 90% and the lower bound of the 95% confidence interval for the proportion of ACPR in subjects treated with DHA/PPQ or ATQ/PG is > 90%, approximately 100 subjects per arm was the target sample size. Although not intended to determine the difference in efficacy between arms, a sample size of 100 per group provided 90% power to detect the difference in efficacy of 95% in one regimen and 80% in another.

### Statistical analysis

The primary analysis was drug efficacy, calculated as the per cent of subjects not requiring alternative therapy during the 42-day course of the study and reported with 95% confidence intervals (95% CI) using the per-protocol dataset per WHO recommendation [17]. Additionally, PCR-corrected efficacy was estimated by Kaplan–Meier survival analysis and the incidence rates of the arms were compared using the log-rank test using R version 4.0.5 [27] and R Studio version 1.4.1106 [28] using the following packages: dplyr (version 1.0.5), survival (version 3.2–10), and survminer (version 0.4.9). To examine differences in parasite drug susceptibilities among 3 regimens, non-parametric Kruskal–Wallis tests were used to compare IC_50_ between groups. The correlation between MQ IC_50_ and *Pfmdr1* copy number was analysed using Spearman’s rank correlation test and the Mann–Whitney test was used to examine the difference in MQ IC_50_ among parasites with < 1.5 and ≥ 1.5 copy numbers. The non-parametric Kruskal–Wallis, Spearman’s rank correlation, and Mann–Whitney tests were performed using Graph-Pad Prism version 6.0 (GraphPad Software, Inc, San Diego, CA, USA).

## Results

**Fig. 2:**
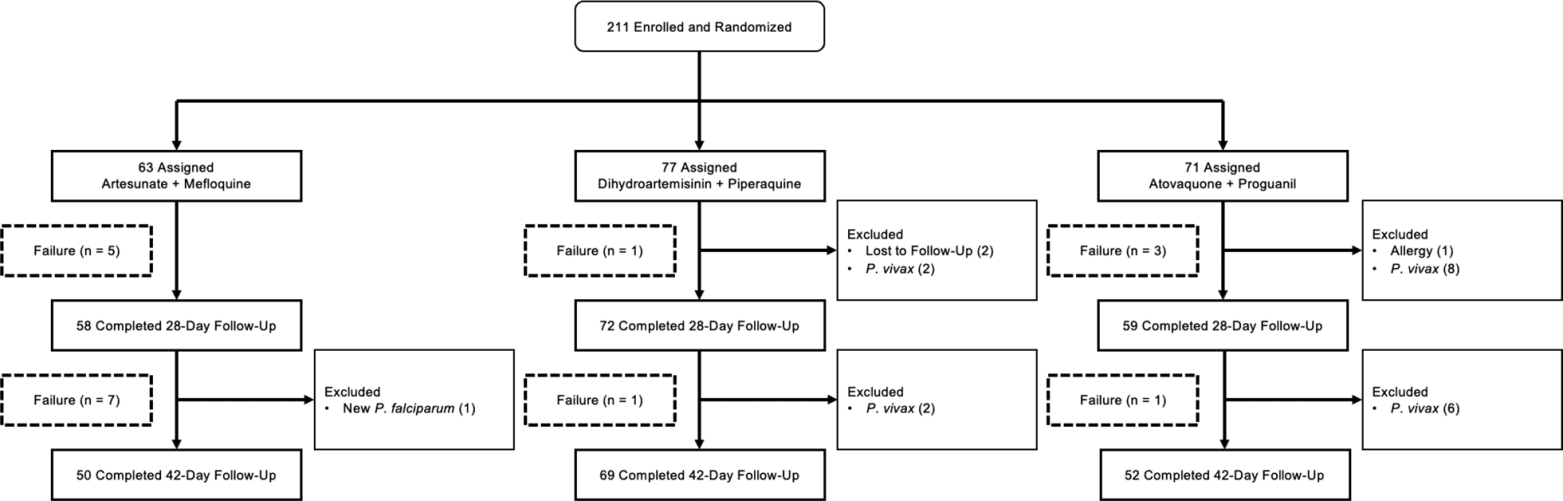
Consort flow diagram

There were 211 subjects meeting criteria for the study who were enrolled from October 2009–November 2011 and randomized to the AS/MQ (63), DHA/PPQ (77), and ATQ/PG (71) arms as in Fig. 2. The target enrolment of 100 subjects per arm was not met due to difficulty in recruiting. Overall, 171 subjects completed the 42-day follow-up, whereas 40 subjects did not—18 (8.5%) due to failures and 22 (10.4%) due to exclusions. Of the subjects excluded from the final analysis, 1 from the AS/MQ arm was excluded due to new *P. falciparum* infection; 6 were excluded from the DHA/PPQ arm due to loss to follow up (n = 2) and *P. vivax* infection (n = 4); and 15 subjects were excluded from the ATQ/PG arm due to allergy (n = 1) and *P. vivax* infection (n = 14). For the primary analysis, 189 (89.6%) were analysed. Baseline characteristics were similar between treatment groups as in Table 1 without significant differences in demographics, initial parasitaemia or clinical presentation. Most subjects were male (79.6%) with a mean age of 25.8 years (range 5–60). Mean axillary temperature was 39.6 °C, mean haematocrit was 40.7%, and the geometric mean parasitaemia on enrolment was 11,873 parasites/µL. Baseline gametocytaemia was detected in 46.9% of subjects.

**Table 1:** Baseline demographics

Parameter	AS/MQ	DHA/PPQ	ATQ/PG	*p*
Total, n	63	77	71	
Male sex, n (%)	50 (79)	60 (78)	58 (82)	0.849
Age, mean (SD), years	25 (13)	26 (12)	26 (13)	0.720
Weight, mean (SD), kg	47 (14)	49 (13)	47 (14)	0.605
Duration of symptoms, mean (SD), days	3 (0.5)	3 (0.6)	3 (0.5)	0.912
Axillary temperature, mean (SD), °C	39.7 (0.9)	39.6 (0.9)	39.6 (0.7)	0.652
Haematocrit, mean (SD)	41 (6.0)	41 (6.5)	40 (6.6)	0.573
Asexual parasitaemia/µL, geomean (5th, 95th percentile)	11,085 (1365, 68,000)	12,364 (1468, 73,200)	12,077 (1870, 69,600)	0.848
Parasite density group, /mL, n (%)				
≥ 1000 and ≤ 10,000	34 (54)	35 (45)	34 (48)	0.594
> 10,000 and ≤ 100,000	29 (46)	42 (55)	37 (52)	
Presence of Pf gametocytes, n (%)^a^	27 (44)	36 (47)	35 (51)	0.756

^a^2 subjects in both the AS/MQ and ATQ/PG arms did not have gametocytaemia data

**Fig. 3:**
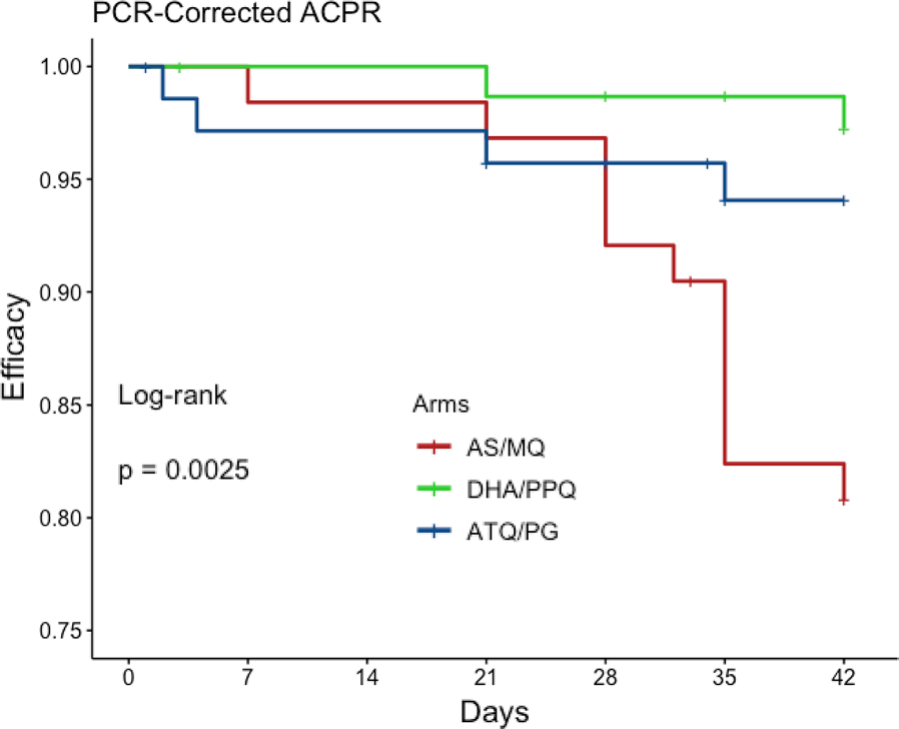
Kaplan Meier survival estimates. Kaplan Meier survival analysis was performed for each of the three study groups to compare parasite-free survival between AS/MQ (red), DHA/PPQ (green) and ATQ/PG (blue). Tic marks on each curve indicate a censored subject

Per-protocol PCR-corrected 42-day ACPR rates (95% CI) for AS/MQ, DHA/PPQ, and ATQ/PG were 80.6% (70.8–90.5%), 97.2% (93.3–100%), and 92.9% (86.1, 99.6%), respectively. Kaplan–Meier estimation of 42-day PCR-corrected ACPR (95% CI) was 80.8% (71.6–91.2%) for AS/MQ, 97.3% (93.6–100%) for DHA/PPQ, and 94.1% (88.6–99.9%) for ATQ/PG (Fig. 3). Rates of 42-day ACPR among the 3 arms differed significantly by the log-Rank test $\left( {p = 0.0025} \right)$. The Day 3 Positivity for the ACT arms was high at 57.9%, suggesting artemisinin resistance (Table 2). Of the 12 failures in the AS/MQ arm, 10 were late clinical failures (LCF) and 2 were late parasitological failures (LPF); both failures in the DHA/PPQ arm were LCF; and of the 4 failures in the ATQ/PG arm, 2 were early treatment failures (ETF) and 2 were LCF (Table 3). The criteria for ETF, LCF, and LPF were described in the 2009 WHO guidance as it describes outcomes for therapeutic efficacy studies followed for 42 days [29].

**Table 2:** Parasite clearance

Day of parasite clearance	AS/MQ n (%)	DHA/PPQ n (%)	ATQ/PG^a^ n (%)	*p*
Before day 3	15 (23.8)	5 (6.5)	4 (5.8)	0.009
On day 3	15 (23.8)	24 (31.2)	22 (31.9)	
After day 3	33 (52.4)	48 (62.3)	43 (62.3)	

^a^1 subject excluded due to drug allergy and another due to day 2 failure in the ATQ/PG arm

**Table 3:** Breakdown of outcomes

Outcome	AS/MQ	DHA/PPQ	ATQ/PG
ACPR	50	69	52
ETF	0	0	2
LCF	10	2	2
LPF	2	0	0
Excluded	1	6	15
Total	63	77	71

The overall mean *Pfmdr1* copy numbers (95% CI) at baseline was 2.2 (2.0–2.4, Table 4). In the AS/MQ arm, treatment failures had higher *Pfmdr1* copy numbers than those with ACPR (3.5 versus 1.8 copies, respectively; $p < 0.00001$ by *t*-test). The *Pfmdr1* copy numbers measured at baseline and on the day of recrudescence are listed for all AS/MQ failures in Additional file 2: Table S1. Interestingly, both DHA/PPQ treatment failures had elevated *Pfmdr1* copy numbers at baseline (3.9 copies), but not at recrudescence (1.0 copies). There was no difference in *Pfmdr1* copy numbers between ACPR and failures in the ATQ/PG arm and all recrudescences were wild-type for cytochrome b mutations.

**Table 4:** Treatment efficacy and *Pfmdr1* copy number

Regimen	Recrudescence	Number of subjects	*Pfmdr1* copy number Day 0 mean (95% CI)	*Pfmdr1* copy number day of recrudescence mean (95% CI)
AS/MQ	Yes	12^a^	3.5 (2.8–4.2)	3.6 (2.4–5.3)
	No	49	1.8 (1.6–2.1)	
DHA/PPQ	Yes	2	3.9	1.0
	No	74	2.2 (1.9–2.4)	
ATQ/PG	Yes	4	2.1	1.6
	No	65	2.2 (1.9–2.5)	

^a^1 subject was censored in the AS/MQ arm due to new infection

**Fig. 4:**
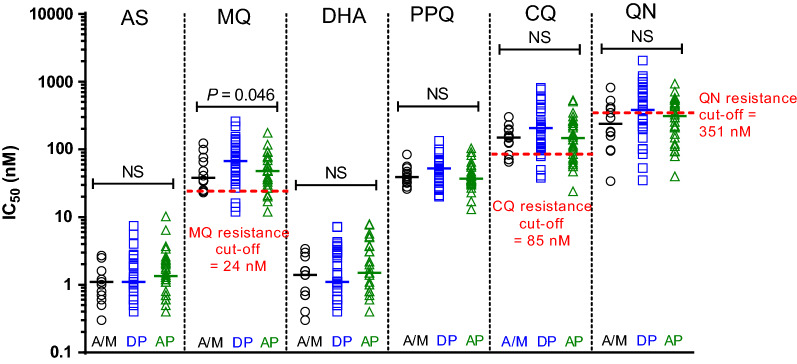
Comparing in vitro parasite drug resistance against common anti-malarials. Samples from 83 subject isolates with > 6400 parasites/µL were run in a classical isotopic *P. falciparum *in vitro drug resistance assay. In vitro resistance was calculated as the 50% inhibitory concentrations (IC_50_) based on serial dilutions of artesunate (AS), mefloquine (MQ), dihydroartemisin (DHA), piperaquine (PPQ), chloroquine (CQ) and quinine (QN). Resistance cut-offs established for the assays where known were used (MQ, CQ and QN). Resistance was compared between the three treatment groups (artesunate–mefloquine (A/M) in black circles; dihydroartemisin-piperaquine (DP) in blue squares, and atovaquone–proguanil (AP) in green triangles

**Fig. 5:**
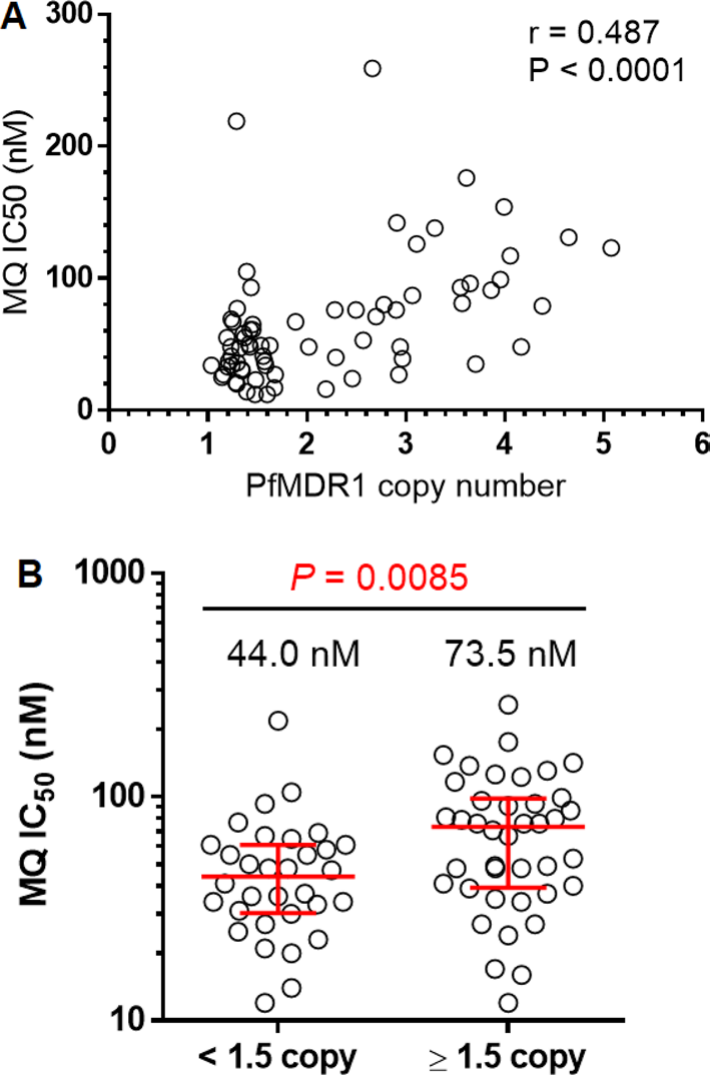
Relationship between mefloquine IC_50_ and *Pfmdr1* status. **A** Correlation between MQ IC_50_ and *Pfmdr1* copy number. **B** Mefloquine IC_50_ was elevated in parasites with ≥ 1.5 copies of *Pfmdr1*

The study was conducted prior to the discovery of currently recognized molecular markers of artemisinin and piperaquine resistance. In vitro drug resistance was assessed using a classical isotopic test for samples with > 6400 parasites/µL as in Fig. 4. The only statistically significant difference between groups was observed for MQ IC_50_ $\left( {p = 0.046} \right)$. The mean IC50 for MQ, CQ, and QN were elevated above their resistance cut-offs for each treatment arm in Fig. 4. Mefloquine resistance correlated with increased *Pfmdr1* copy number ($r = 0.487$, $p < 0.0001$) and MQ IC_50_ was higher in those with ≥ 1.5 copies (73.5 nM) than those with < 1.5 copies (44.0 nM) at Day 0 $\left( {p = 0.0085} \right)$, as in Fig. 5.

## Discussion

In this 2009–2011 therapeutic efficacy study in Oddar Meanchey, PCR-corrected failure rates for AS/MQ, ATQ/PG and DHA/PPQ were 19%, 6%, and 3%, respectively. The Day 3 Positivity for the 2 ACT arms was nearly 60%, suggesting a high level of clinical artemisinin resistance. However in vitro parasite resistance assays on a subset of samples did not indicate significant artemisinin resistance. The WHO recommends changing therapy when failure rates exceed 10% and instituting containment measures when Day 3 positivity exceeds 10% [30]. Data from the study was provided to CNM at the time and used to support changes to therapy including the transition to DHA/PPQ. Overall, there were 2.2 copies of *Pfmdr1* on average, consistent with previous work by NAMRU-2 which estimated mean copy number at this site at ~ 2. Further, there was both in vitro evidence of MQ resistance and unacceptably high rates of AS/MQ clinical failures at this site. Treatment failures in the AS/MQ arm had higher baseline *Pfmdr1* copy numbers than those with ACPR (3.5 versus 1.8 copies, respectively). Both DHA/PPQ treatment failures had baseline *Pfmdr1* amplification, but lost amplification at recrudescence. Baseline gametocytaemia, important for mosquito transmission, was the highest reported among clinical studies in Cambodia (47%) and higher than the global average of 12% in a meta-analysis [31]. Some factors predictive of baseline gametocytaemia in Asia were present in this study (age, male gender, and low density of parasitaemia) but not others (afebrile disease and anaemia) [31]. This also supports the notion of clinically smouldering infections in the present study indicative of partially treated and/or resistant infections.

This study occurred during the national transition from AS/MQ and helps bridge the historic gap in understanding the transition to full-blown DHA/PPQ failures that occurred rapidly between 2008 and 2014 on the Thai–Cambodian border. Table 5 summarizes clinical studies of uncomplicated falciparum malaria in Cambodia from 2001 to 2018. A brief review of historic and interim findings related to resistance to the drugs used in the present study is incorporated with a discussion of the findings. While some of the commonly used molecular markers of resistance used today were not available, there was clear elevation of *Pfmdr1* copy number among subjects with AS/MQ failures, as well as elevated MQ IC_50_s in those with elevated copy numbers.

**Table 5:** Clinical studies of uncomplicated *P. falciparum* malaria in Cambodia, 2001–2018

Study	Date	Province	Regimen, n	Baseline gametocyte	Day 3 positive	Clearance (h)	*PfK13*	*PfMdr1*	*PfPlasmepsin*	PCR-ACPR (days)
Denis et al. 2006^a^	2001	Battambang	AS/MQ, 50							96% PP (28)
	2003	Battambang	AS/MQ, 55							92% PP (28)
	2002	Pailin	AS/MQ, 81							86% PP (28)
	2004	Pailin	AS/MQ, 90		10%			1.5 copies Overall		79% PP (42)
	2002	Pursat	AS/MQ, 83							93% PP (28)
	2004	Pursat	AS/MQ, 85							93% PP (28)
	2003	Oddar Meanchey	AS/MQ, 92							98% PP (28)
	2002	Preah Vihear	AS/MQ, 36							97% PP (28)
	2004	Preah Vihear	AS/MQ, 85							100% PP (42)
	2001	Kratie	AS/MQ, 50							100% PP (28)
	2003	Kratie	AS/MQ, 66							100% PP (28)
	2002	Ratanakiri	AS/MQ, 75							100% PP (28)
	2004	Ratanakiri	AS/MQ, 80							100% PP (42)
	2003	Kampong Speu	AS/MQ, 97							97% PP (28)
Rogers et al. 2009^b^	2006–2008	Kampot	AS/MQ, 151		11%			2.7 copies fail 1.9 copies ACPR		81% KM (42)
Noedl et al. 2008 Noedl et al. 2010^c^	2006–2007	Battambang	AS, 74		22%	PCT = 58		1.1 copies overall		94% KM (28)
			QN + TET, 37		34%	PCT = 78				100% KM (28)
Rueangweerayut et al. 2012^d^	2007–2008	Pailin	AS/PYR, 140		37%	PCT = 64				90% KM (42)
			AS/MQ, 71		38%	PCT = 64				100% KM (42)
Dondorp et al. 2009^e^	2007–2008	Pailin	AS/MQ, 20	30%	55%	PCT = 72		5% amplified		95% ITT (63)
			AS, 20	25%		PCT = 84				70% ITT (63)
Leang et al. 2013^f^	2008–2009	Pailin	DHA/PPQ, 59		26%			17% amplified		90% KM (42)
	2009–2010	Pailin	DHA/PPQ, 41		33%			24% amplified		92% KM (42)
	2010–2011	Pailin	DHA/PPQ, 30		45%			16% amplified		76% KM (42)
	2008	Pursat	DHA/PPQ, 86		8%			32% amplified		99% KM (42)
	2010	Pursat	DHA/PPQ, 64		10%			25% amplified		90% KM (42)
	2009	Preah Vihear	DHA/PPQ, 60		5%					100% KM (42)
	2009–2010	Ratanakiri	DHA/PPQ, 59		0%					100% KM (42)
	2010	Ratanakiri	DHA/PPQ, 61		0%					100% KM (42)
Bethell et al. 2011^g^	2008–2009	Battambang	AS2, 75	13%	49%	PCT = 74				88% PP (42)
			AS4, 40	10%	46%	PCT = 78				90% PP (42)
			AS6, 28	21%	48%	PCT = 78				79% PP (42)
Amaratunga et al. 2012^h^	2009–2010	Pursat	AS/MQ, 180	16%		PC_1/2_ = 5.9 PCT = 78				
This study^i^	2009–2011	Oddar Meanchey	AS/MQ, 63	44%	52%			3.5 copies fail 1.8 copies ACPR		81% KM (42)
			DHA/PPQ, 77	47%	62%			3.9 copies fail 2.2 copies ACPR		97% KM (42)
			ATQ/PG, 71	51%	62%			2.1 copies fail 2.2 copies ACPR		94% KM (42)
Lon et al. 2014^j^	2010–2011	Oddar Meanchey	DHA/PPQ × 2, 8	13%	50%	PCT = 80	82% C580Y	1.2 copies fail 1.1 copies ACPR		75% PP (42)
			DHA/PPQ × 3, 12	42%	42%	PCT = 68				82% PP (42)
Leang et al. 2015^k^	2011–2013	West	DHA/PPQ, 147		31%		87% C580Y	16% amplified		85% PP (42)
		East	DHA/PPQ, 278		17%		23% C580Y	21% amplified		98% PP (42)
Ashley et al. 2014^l^	2011–2013	Pailin	AS4 + DHA/PPQ, 100	19%	66%	PC_1/2_ = 6.1	~ 75% C580Y		~ 50%	98% PP (42)
		Pursat	AS4 + DHA/PPQ, 120	18%	60%	PC_1/2_ = 5.6				
		Preah Vihear	AS2/4 + DHA/PPQ, 120	5%	18%	PC_1/2_ = 3.0	Diverse		Mostly WT	
		Ratanakiri	AS2/4 + DHA/PPQ, 120	6%	7%	PC_1/2_ = 3.0	Mostly WT		100% WT	
Amaratunga et al. 2016^m^	2012–2013	Pursat	DHA/PPQ, 110	17%	61%	PC_1/2_ = 6.1	77% Mutant	0% amplified fail 11% amplified ACPR		63% KM (63)
		Preah Vihear	DHA/PPQ, 65	9%	26%	PC_1/2_ = 3.0	34% mutant			85% KM (63)
		Ratanakiri	DHA/PPQ, 66	2%	3%	PC_1/2_ = 2.4	11% mutant			98% KM (63)
Saunders et al. 2014 Spring et al. 2015^n^	2012–2014	Oddar Meanchey	DHA/PPQ, 51	10%	65%	PC_1/2_ = 6.4 PCT = 80	65% C580Y 31% R539T			58% mITT (42)
			DHA/PPQ + PQ, 50	8%	42%					50% mITT (42)
Leang et al. 2016^o^	2014–2015	Pailin	AS/PYR, 55	0%	13%		96% C580Y			84% KM (42)
		Pursat	AS/PYR, 60	8%	44%					90% KM (42)
		Battambang	AS/PYR, 8	0%	25%					100% KM (42)
Wojnarski et al. 2019^p^	2014–2015	Oddar Meanchey	ATQ/PG + PQ, 79	21%	42%	PC_1/2_ = 5.6	100% C580Y			88% KM (42)
			AS + ATQ/PG + PQ, 78		42%	PC_1/2_ = 5.9	99% C580Y			91% KM (42)
		Kratie	ATQ/PG + PQ, 24	19%	17%	PC_1/2_ = 5.6	91% C580Y			100% KM (42)
			AS + ATQ/PG + PQ, 24		4%	PC_1/2_ = 5.3	86% C580Y			94% KM (42)
Van der Pluijm et al. 2019 Van der Pluijm et al. 2020^q^	2015–2018	Pailin	DHA/PPQ + PQ, 9	41%	72%	PC_1/2_ = 7.2	97% C580Y	0% amplified	69%	52% KM (42)
			DHA/PPQ + MQ + PQ, 11							100% KM (42)
			AS/MQ + PQ, 22							100% KM (42)
			DHA/PPQ + MQ + PQ, 22							100% KM (42)
		Pursat	DHA/PPQ + PQ, 8		46%	PC_1/2_ = 6.3	89% C580Y		79%	25% KM (42)
			DHA/PPQ + MQ + PQ, 11							100% KM (42)
			AS/MQ + PQ, 48							100% KM (42)
			DHA/PPQ + MQ + PQ, 45							100% KM (42)
		Preah Vihear	AS/MQ + PQ, 3		14%	PC_1/2_ = 5.2	100% C580Y		100%	100% KM (42)
			DHA/PPQ + MQ + PQ, 4							100% KM (42)
		Ratanakiri	DHA/PPQ + PQ, 44	16%	52%	PC_1/2_ = 7.0	85% C580Y		71%	73% KM (42)
			DHA/PPQ + MQ + PQ, 46							100% KM (42)

*PP* per-protocol, *QN* quinine, *TET* tetracycline, *PYR* pyronaridine, *PQ* primaquine, *PCT* parasite clearance time, *PC*_*1/2*_ parasite clearance half-life, *WT* wild-type, *ITT* intention-to-treat, *mITT* modified intention-to-treat, *KM* Kaplan–Meier

^a^Report of 14 therapeutic efficacy studies [9]; Day 3 Positive and median *Pfmdr1* copies from Alker et al. [40]

^b^Therapeutic efficacy study reporting mean *Pfmdr1* copies; failures had 3.6 copies at recrudescence [10]

^c^Randomized controlled trial reporting median PCT and *Pfmdr1* copies [64, 65]

^d^Phase 3, randomized controlled trial reporting median PCT [66]

^e^Randomized controlled trial with another site in Wang Pha, Thailand; reporting median PCT [41]

^f^Report of 8 therapeutic efficacy studies [42]

^g^Randomized controlled trial comparing 2, 4, and 6 mg/kg AS; reporting median PCT [67]

^h^Parasite clearance rate study reporting geometric mean PC_1/2_ [33]

^i^Randomized controlled trial; mean *Pfmdr1* copies at recrudescence were 3.6, 1.0, and 1.6 for AS/MQ, DHA/PPQ, and ATQ/PG, respectively

^j^Randomized controlled trial comparing 2- versus 3-day regimens of DHA/PPQ, reporting median PCT and *Pfmdr1* [47]; *PfK13* mutations were essentially at fixation [35]

^k^Therapeutic efficacy study; Western refers to Battambang, Pursat, Kampot, and Kampong Speu; Eastern refers to Kratie, Preah Vihear, Ratanakiri, and Kampong Thom [13]

^l^TRACI: parasite clearance rate study of AS 2 or 4 mg/kg with DHA/PPQ, reporting median PC_1/2_ [36]; *PfK13* and *Pfplasmepsin* status reported in van der Pluijm et al. [43]

^m^Therapeutic efficacy study reporting median PC_1/2_ [37]

^n^Randomized controlled trial reporting median PC_1/2_ and PCT [12, 35]; Gametocytemia reported in Lin et al. [61]

^o^Therapeutic efficacy study [68]

^p^Randomized controlled trial reporting median PC_1/2_ [51]

^q^TRACII: randomized controlled trial; gametocytemia for Pailin and Pursat refer to the 17 subjects receiving DHA/PPQ [43, 44]

### Artemisinin resistance

Artemisinin resistance is defined by parasite clearance half-life (PC_1/2_) ≥ 5 h, but Day 3 Positivity ≥ 10% is considered a useful clinical surrogate [32]. Day 3 positivity increased rapidly (10 to 55%) in Pailin in AS/MQ studies from 2004 to 2008. In DHA/PPQ studies from 2008 onward, Day 3 positivity was generally higher in Western than Eastern provinces. In that period, the rate in Pailin increased from 26 to 72% while remaining relatively constant (between 42 and 65%) in Oddar Meanchey. Parasite clearance half-life was 5.9 h in a 2009–2010 AS/MQ parasite clearance rate study in Pursat [33] and was generally longer in Western than Eastern Cambodia. Since 2014, average PC_1/2_ has consistently exceeded 5 h throughout Cambodia.

Mutations in *Pfkelch 13* (*k13*) are newer molecular markers of artemisinin resistance [34] and with the exception of Eastern Cambodia, have essentially been at fixation since 2010. The C580Y single nucleotide polymorphism (SNP) was higher in Western than Eastern provinces, but became the dominant *k13* allele throughout Cambodia by 2014–2015. In a 2010 Oddar Meanchey study, all failures were C580Y, and 3 year later, all failures were either C580Y or R539T [35]. Several *k13* mutations were strongly associated with prolonged PC_1/2_ in Pailin and with recrudescence throughout Cambodia [36, 37]. Near simultaneous studies found C580Y associated with high Day 3 positivity, prolonged PC_1/2_, and post-treatment gametocytaemia in Oddar Meanchey [35] and with treatment failures throughout Cambodia [13]. In this 2009–2011 study, Day 3 positivity ranged from 51 to 62% suggesting a level of artemisinin resistance not previously appreciated at the time in Northern Cambodia. Therefore, the difference in efficacy between AS/MQ and DHA/PPQ (79% and 97%, respectively) probably reflects differing partner drug susceptibility. Resistance to AS and DHA as measured by IC_50_ was relatively low, though it is unclear how predictive these were in the isotopic assays used at the time. In the interval, ring-stage survival assays for the artemisinins have been developed and are more widely used [38].

### Mefloquine resistance

Mefloquine resistance was reported shortly after its introduction on the Thai–Cambodian border [39]. Amplification of *Pfmdr1* is a molecular marker of MQ resistance, which leads to higher expression of the Multidrug Resistance Protein 1 (MDR1) pump and higher MQ IC_50_ [15, 25]. An early AS/MQ efficacy study found an association between high copy numbers and treatment failures [40] and another efficacy study reported a rise in copy numbers in recrudescent compared to baseline samples (3.6 versus 2.7, respectively) [10]. However, a later AS/MQ randomized study found no association with *Pfmdr1* copy numbers and treatment failure [41]. During the transition to DHA/PPA, therapeutic efficacy studies from 2008 to 2010 surprisingly revealed increasing MQ IC_50_, though 17 of 18 failures were *Pfmdr1* deamplified [42]. This discrepancy was seemingly resolved in 2012–2013 efficacy studies in 3 provinces where all 48 failures were *Pfmdr1* deamplified and had lower MQ IC_50_ than those with ACPR [37]. These studies suggest that PPQ exposure drives *Pfmdr1* deamplification but additional factors may play a role in MQ resistance. By the time of the 2015–2018 Tracking Resistance to Artemisinin Collaboration 2 (TRACII) study, no isolates were *Pfmdr1* amplified following the prior withdrawal of AS/MQ as first line agent for several years [43, 44]. In the present study, high baseline *Pfmdr1* copy numbers were observed, especially among AS/MQ failures. Both DHA/PPQ failures had deamplified copy numbers at recrudescence (from 3.9 to 1 copies), suggesting either selection of a minor population or de novo deamplification after exposure to PPQ.

### Piperaquine resistance

Piperaquine, a bisquinoline similar to chloroquine was used as monotherapy in Pailin and elsewhere for long periods since the 1990s [42]. Piperaquine resistance appears to be multifactorial with multiple molecular markers identified and confirmed clinically over the past decade. The mutant *Pfcrt* haplotype *CVIET*, associated with CQ and PPQ resistance, likely originated in Southeast Asia in the 1970s or earlier [45, 46]. All tested isolates in the 2008–2010 therapeutic efficacy studies and the 2010–2011 Oddar Meanchey study were CVIET [42, 47]. Deamplification of *Pfmdr1* is another marker of PPQ resistance, seen in 17 of 18 failures in a study from 2008 to 2010 and all failures in a 2011–2013 study [13, 42]. The former study did not see a rise in PPQ IC_50_ over time and the latter study did not correlate copy numbers to clinical outcomes. Significant clinical failure of DHA/PPQ was first reported in a 2012–2014 Oddar Meanchey study that was halted [12]. An ex vivo susceptibility study determined that DHA/PPQ clinical failures were preceded by increases in PPQ IC_90_ and decreases in *Pfmdr1* copy number and MQ IC_50_ [48]. By 2012, a study found that recrudescent parasites had higher PPQ IC_50_ and lower MQ IC_50_ than those with ACPR; none of the failures were *Pfmdr1* amplified [37]. At the onset of the 2015–2018 TRACII study, where all isolates were *Pfmdr1* deamplified, DHA/PPQ was still utilized [43]. That study confirmed high rates of failure of DHA/PPQ, associated with *Pfplasmepsin 2/3* amplification and 4 new *Pfcrt* mutations (T93S, H97Y, F145I, I218F) [43]. Retrospective analysis of the 2011–2013 TRACI study found that over half of isolates in Western Cambodia were *Pfplasmepsin 2/3* amplified. In 2011, the *Pfplasmepsin 2/3* amplification and *Pfcrt* mutations were rarely found in Eastern Cambodia, but have since expanded to be dominant throughout Cambodia as observed in the TRACII study [43]. The present study had only 2 DHA/PPQ failures, but to our knowledge, deamplification of *Pfmdr1* in paired baseline and recrudescent samples has not been previously described.

### Counterbalance of PfMDR1 and PfCRT mutations

The differential binding of quinoline drugs and opposing actions of the MDR1 and Chloroquine Resistance Transporter (CRT) pumps affect parasite susceptibility to anti-malarials [49]. The CRT pump transports CQ out of the digestive vacuole, where it interferes with haem detoxification; the MDR1 pump transports MQ into the digestive vacuole and away from a cytosolic site of action [49]. Chloroquine resistance arose along the Thai-Cambodian border in the late 1950s [6], associated with the mutant *Pfcrt* haplotype *CVIET* (codons 72–76) [45]. A genetic engineering study demonstrated that *Pfmdr1* haplotypes modulate susceptibilities to different anti-malarials in the presence of *CVIET* [49]. The *Pfmdr1 NF* haplotype (wild-type N86, mutant Y184F) decreases susceptibility to CQ and PPQ in presence of *Pfcrt* CVIET [49] and is associated with MQ resistance [40]. The *Pfmdr1 NY* haplotype (wild-type for both N86 and Y184) is associated with MQ sensitivity [40]. In a retrospective analysis of a 2004 AS/MQ Pailin study, most parasites were either *Pfmdr1 NF* (76%) or *NY* (17%) haplotypes [40]. Both *Pfmdr1* haplotypes also co-occurred with *Pfmdr1* amplification, though they were more common among NY isolates [49]. From 2009 to 2013, coinciding with the transition to DHA/PPQ, northern Cambodian isolates saw declining *Pfmdr1* amplification and increasing Y184F mutations [11]. In another Oddar Meanchey study done around the same time as this study, all isolates were CVIET and 19 of 20 were *Pfmdr1 NF* and mostly deamplified [47]. Not surprisingly, the mean CQ IC_50_ in this study was above 85 nM, suggesting baseline CQ resistance.

### Atovaquone–proguanil resistance

Since 2006, ATQ/PG has been used in WHO-sponsored containment efforts along the Thai border and remains effective in preventing and treating multidrug-resistant *P. falciparum* [50, 51]. Due to its primary activity against the causal liver stage [52], resistance could quickly develop when used against the blood-stage or for mass drug administration [53]. Atovaquone inhibits *P. falciparum* cytochrome bc_1_ complex and 3 cytochrome b (*Pfcytb*) SNPs (Y268N, Y268S, Y268C) are strongly associated with in vitro resistance and late clinical failure [50, 52]. Though mutations in *Pfcytb* appear to occur frequently, they may come at a fitness cost and the inability to persist in the population [50]. Recent studies in Oddar Meanchey suggest a low level of ATQ resistance. All failures in the 2012–2014 DHA/PPQ Oddar Meanchey study were wild-type *Pfcytb* 268 and sensitive to ATQ in vitro [52]. All parasites in a 2014–2015 Oddar Meanchey and Kratie study were *Pfcytb* wild-type at baseline, but one late failure developed the Y268C mutation on day 28 [54]. The partner drug, proguanil, is biotransformed into the dihydrofolate reductase inhibitor cycloguanil (CYC) by CYP2C19. It is believed that PG has greater synergy with ATQ [55, 56]. One reason is that a high level of CYC resistance has been long established in Southeast Asia, likely mediated by the *Pfdhfr* SNPs S108T and A16V [51, 54, 56]. Another reason is the sizeable portion of Asians who may be CYP2C19 poor metabolizers, which result in lower CYC levels [50, 56]. Interestingly, while all 14 failures in the 2014–2015 study lacked *Pfdhfr* S108T and A16V mutations, they were all quadruple mutants with S108N, N51I, C59R, and I164L SNPs—previously associated with pyrimethamine resistance but is now thought to convey CYC resistance [54]. Further, among 17 case reports with known *Pfdhfr* status, failures within 3 days tended to occur among wild-type while failures at 7 days or later occurred among triple mutants (S108N, N51I, C59R), suggesting a possible role of this haplotype in ATQ/PG failures [50]. None of the parasites in the present study had *Pfcytb* mutations at baseline, including the 4 recrudescences in the ATQ/PG arm. The declining efficacy from the present study (94%) compared to the 2014–2015 study (88%) suggests increasing resistance from factors that need further exploration.

### Drug pressure and evolution

The C580Y *Pfk13* mutation has been present in Western Cambodia since at least 2001 and a distinct C580Y haplotype, *KEL1*, arose as early as 2007 in Western Cambodia, dominating that region by 2012–2013 [34]. *Pfplasmepsin 2/3* amplifications in Cambodia likely arose from the *PLA1* haplotype as early as 2002 [57]. In 2008, Western Cambodia saw both the adoption of DHA/PPQ as first-line therapy and the merger of *KEL1/PLA1* haplotypes associated with DHA/PPQ failures [57]. This co-lineage has since spread outside Cambodia and by 2016–2017 became dominant in several Southeast Asian nations [58]. Interestingly, *KEL1* was associated with *Pfmdr1* amplifications prior to 2008, but by 2013 had mostly deamplified and rapidly gained *Pfplasmepsin 2/3 amplifications* in Western Cambodia [57]. Newer molecular markers for anti-malarial failure are being identified, such as the new *Pfcrt* mutations (T93S, H97Y, F145I, I218F) [43, 58], the exonuclease E415G mutation [59, 60], and the quadruple *Pfdhfr* mutations (S108N, N51I, C59R, and I164L) [54]. These mutations occurred on a genetic background of *CVIET*, *KEL1*, and *PLA1* and are fuelled by drug pressure.

## Limitations

This study was completed in 2011 and no stored biospecimens were available for analysis of drug resistance markers that have since been discovered. The study was open label, but efforts were made to prevent randomization bias, and subjects received directly observed therapy. Twenty-two subjects (10%) were excluded from final analysis of the primary endpoint due to new *P. falciparum* infection (1), loss of follow up (2), allergy (1), or *P. vivax* infection (18). Baseline levels of anti-malarials were not drawn, which are indicators of private sector use of anti-malarials, which may fuel the development of resistance. However, detectable PPQ levels at baseline are not always associated with clinical outcomes; recrudescence was associated in one study [37] but not another [35]; baseline gametocytaemia was associated in one study [61] but not another [43]. Many biomarkers for anti-malarial resistance were not yet validated or commonly available at the time of the study. *Pfk13* mutations were described in 2014 [34] and PC_1/2_ estimation was described in 2011 [62]. The isolates were not tested for multiple markers of resistance to include *Pfcrt CVIET*, though this is now recognized as the most common haplotype in the region [11, 47, 49]. The newer quadruple *Pfcrt* mutations were not described until 2019 [43]. *Pfplasmepsin 2/3 amplifications* were associated with treatment failures of DHA/PPQ in 2017 [59, 63] and there is evidence that the E415G substitution of an exonuclease is a potential biomarker for PPQ resistance and failure [59, 60].

## Conclusion

This 2009–2011 study was the first in Oddar Meanchey conducted during the transition to DHA/PPQ as first line therapy. This preceded a 2010–2011 study which saw 25% failure rate [47] and a 2012–2014 study which saw 50% failure rate [12, 35]. In the interim, a decision was first made to adopt DHA/PPQ based on results from this study and others. However, in a short period of time, parasites rapidly acquired genetic mutations which conveyed PPQ resistance. The present declining efficacy of DHA/PPQ mirrors the declining copy numbers of *Pfmdr1*. Continued efforts to maximize non-pharmacologic therapies, use directly observed and publicly provided anti-malarial therapy, surveillance studies to include molecular markers, and eradication of *P. falciparum* are needed in the region.

## Supplementary Information

**Additional file 1.** Protocol: efficacy of three standard therapies for uncomplicated *P. falciparum* malaria in Cambodia, version 1.4.

**Additional file 2: Table S1.** *Pfmdr1* copy numbers for AS/MQ failures.

## Data Availability

The datasets used during this study are available from the corresponding author on reasonable request. The data may be shared with the Worldwide Antimalarial Research Network (WWARN). The protocol is available as an Additional file 1.

## Abbreviations

ACPR: Adequate clinical and parasitological response
ACT: Artemisinin-based combination therapy
AS/MQ: Artesunate–mefloquine
AS: Artesunate
ATQ: Atovaquone
ATQ/PG: Atovaquone–proguanil
CNM: Cambodian National Centre for Parasitology, Entomology, and Malaria Control
CQ: Chloroquine
CRT: Chloroquine Resistance Transporter
CYC: Cycloguanil
DHA: Dihydroartemisinin
DHA/PPQ: Dihydroartemisinin–piperaquine
ETF: Early treatment failure
IC_50_: Half-maximal inhibitory concentration
K13: *PfKelch 13*
LCF: Late clinical failure
LPF: Late parasitologic failure
MDR1: Multidrug Resistance Protein 1
MQ: Mefloquine
NAMRU-2: U.S. Naval Medical Research Unit 2
PC_1/2_: Parasite clearance half-life
PG: Proguanil
PPQ: Piperaquine
RFLP: Restriction fragment length polymorphism
SNP: Single nucleotide polymorphism
TRAC: Tracking Resistance to Artemisinin Collaboration
QN: Quinine
WWARN: Worldwide Antimalarial Resistance Network
WHO: World Health Organization
