# Supplementary material for: Efficacy of three anti-malarial regimens for uncomplicated Plasmodium falciparum malaria in Cambodia, 2009–2011: a randomized controlled trial and brief review
Source: Malar J. 2022 Sep 7;21:259. doi: 10.1186/s12936-022-04279-3 (PMC9450427; doi:10.1186/s12936-022-04279-3)
Supplement: Supplementary file 1 — Additional file 1. Protocol: efficacy of three standard therapies for uncomplicated P. falciparum malaria in Cambodia, version 1.4. [file 12936_2022_4279_MOESM1_ESM.doc]

# I. COVER PAGE

**A. Protocol Number:** 2009 – NAMRU2.2009.0001

**B. Protocol Title**: Efficacy of three standard therapies for uncomplicated *P. falciparum* malaria in Cambodia

**C. Relevant NAMRU-2 Work Unit**: GEIS (D0016)/BEP (D0501)

**D. Principal Investigator**: Dr. Lek Dysoley, National Malaria Center, Phnom Penh, Cambodia

**E. NAMRU-2 Submitting Investigator/IRB Certification Number:** CAPT William O. Rogers/IRB# 0702140230

**F. Co-Investigators:**

**National Malaria Center, Phnom Penh (FWA#00010451)**

Dr. Duong Socheat

**Institute Pasteur de Cambodge, Phnom Penh** (FWA#00009898)

Mrs. Sem Rithy

Mr. Phektra Chim

**G. Research Location:** Phnom Penh, Oddarmenchey

**H. Lead Agency:** National Malaria Center, Phnom Penh. DOD lead agency is US NAMRU-2

**I. Collaborating Domestic Institutions:** None

**J. International Ethical Approval:** National Ethics Committee, Cambodia

**K. Proposed Start and End Dates:** 01 August 2009 through 31 October 2012

**L. Projected/Estimated Total Number of Subjects:** 300

**M. Inclusions:** Individuals of age ≥ 5 years found to be microscopically positive for *P. falciparum* malaria

**N. Exclusions:** Individuals with severe malaria requiring emergency treatment or referral; mixed species malaria infection; allergy or contraindication to any study drug; pregnant women or nursing mothers; unwillingness or incapacity to give informed consent.

**O. Anticipated Risks**:

1. Risk of treatment failure

2. Risk of known side effects of treatment with the three standard anti-malarial drug regimens

3. Risk of discomfort or infection from fingerprick blood sampling or venipuncture.

**P. Proposed Risk Reduction Methodologies and Provisions:**

1. The outcome of treatment will be followed closely with periodic malaria smears and recurrence of parasitemia will be treated with quinine-tetracycline, a regimen to which resistance is negligible.

2. Women of child-bearing age will have a pregnancy test before enrollment to reduce the risk of side effects in pregnancy. Mefloquine-artesunate and piperaquine-DHA are currently recommended by the Cambodian Ministry of Health for uncomplicated *P. falciparum* malaria, and Malarone is approved by the U.S. Food and Drug Administration for prevention and treatment of malaria and currently recommended by the World Health Organization for mass screening and treatment programs in Cambodia.

3. Finger prick and venipuncture will be conducted by well-trained personnel with aseptic technique and sterile, disposable lancets or needles to minimize the risk of infection and bruising.

**Q. Medical Monitor**

Dr. Sinuon Muth, National Malaria Center, Phnom Penh

**R. Protocol Abstract:**

Malaria is a critical infectious disease. Control of malaria is made more difficult by the ongoing spread of drug resistance. There is emerging evidence of resistance to mefloquine-artesunate in a few sites in Cambodia, and a containment zone encompassing several provinces has been established to prevent spread of resistant strains. Ongoing monitoring of the spread of drug resistance is important in the design of control and containment programs. Three anti-malarial regimens are currently recommended by the National Malaria Center, mefloquine-artesunate, piperaquine-DHA, and Malarone (atovaquone-proguanil). It is important to know whether mefloquine-artesunate remains effective outside the containment zone and whether alternative regimens are also effective. We will therefore measure the efficacy of each of these regimens in Oddarmeanchey provine, an area outside of, but adjacent to, the containment zone. We will use a standard WHO protocol to assess in vivo efficacy of anti-malarials using directly observed therapy and a 42 day follow-up period to detect recurrent parasitemia. We will use PCR correction to discriminate treatment failure from re-infection, and will attempt to identify risk factors for treatment failure.

[I. COVER PAGE 1](#__RefHeading___Toc242351255)

[Record of Changes to Protocol 5](#__RefHeading___Toc242351256)

[PART III: SIGNATURES AND INVESTIGATOR ASSURANCE AGREEMENT 8](#__RefHeading___Toc242351257)

[COMMANDING OFFICER SIGNATURE PAGE 10](#__RefHeading___Toc242351258)

[PART IV: PROTOCOL CRITICAL ELEMENTS 11](#__RefHeading___Toc242351259)

[A. SCIENTIFIC BACKGROUND AND OBJECTIVES 11](#__RefHeading___Toc242351260)

[1. Background 11](#__RefHeading___Toc242351261)

[2. Objectives and Hypotheses 12](#__RefHeading___Toc242351262)

[B. EXPERIMENTAL METHODS 13](#__RefHeading___Toc242351263)

[1. Experimental procedures and rationale 13](#__RefHeading___Toc242351264)

[a. Justification for the use of human subjects, including children 13](#__RefHeading___Toc242351265)

[b. Study Location 13](#__RefHeading___Toc242351266)

[c. Volunteer recruitment and enrolment 13](#__RefHeading___Toc242351267)

[d. Inclusion and exclusion criteria 14](#__RefHeading___Toc242351268)

[e. Anti-malarial therapy 16](#__RefHeading___Toc242351269)

[f. Source of Study Drugs 17](#__RefHeading___Toc242351270)

[f. Laboratory procedures 17](#__RefHeading___Toc242351271)

[2. Sample Size 20](#__RefHeading___Toc242351272)

[3. Data Analysis 20](#__RefHeading___Toc242351273)

[4. Required equipment and supplies 21](#__RefHeading___Toc242351274)

[5. Budget 21](#__RefHeading___Toc242351275)

[C. ORGANIZATION OF RESEARCH EFFORT 22](#__RefHeading___Toc242351276)

[1. Duties and Responsibilities of Investigators and Medical Monitor 22](#__RefHeading___Toc242351277)

[2. Multicenter Organizational Plan for IRB Review and Approval 22](#__RefHeading___Toc242351278)

[3. Technology Transfer/Capacity Building/Sample Sharing Plan 22](#__RefHeading___Toc242351279)

[D. RISKS AND BENEFITS FOR SUBJECTS 22](#__RefHeading___Toc242351280)

[1. Risks 22](#__RefHeading___Toc242351281)

[Treatment failure 23](#__RefHeading___Toc242351282)

[Side effects of standard anti-malarial drugs 23](#__RefHeading___Toc242351283)

[Side effects of blood sampling 23](#__RefHeading___Toc242351284)

[Social or economic risks 23](#__RefHeading___Toc242351285)

[2. Benefits 24](#__RefHeading___Toc242351286)

[3. Confidentiality and Sample Storage 24](#__RefHeading___Toc242351287)

[4. Special Risks to Pregnant or Potentially Pregnant Women Volunteers 24](#__RefHeading___Toc242351288)

[5. Safety Precautions and Emergency Procedures 24](#__RefHeading___Toc242351289)

[6. Assessment of Sufficiency of Plans to Deal with Untoward Events or Injuries 24](#__RefHeading___Toc242351290)

[7. Qualification of Medical Monitor and Medical Support Personnel 24](#__RefHeading___Toc242351291)

[E. DESCRIPTION OF THE SYSTEM FOR MAINTENANCE OF RECORDS 25](#__RefHeading___Toc242351292)

[1. Experimental Data 25](#__RefHeading___Toc242351293)

[2. Regulatory File 25](#__RefHeading___Toc242351294)

[3. Individual Medical Records 25](#__RefHeading___Toc242351295)

[Figure 1. Malaria Containment Zones. 26](#__RefHeading___Toc242351296)

[Appendix A. Case Report Form 27](#__RefHeading___Toc242351297)

[Appendix B 39](#__RefHeading___Toc242351298)

## Record of Changes to Protocol

In response to review by the NAMRU-2 Scientific Advisory Board, 09JUN09 the following minor corrections have been made:

p.42 (ICF) different signs have been used to mark the two different footnotes.

p.42 (ICF) The lower age limit on the ICF is corrected to 5 years, to make it consistent with the text of the protocol.

p. 42 (ICF) The lower age limit requiring assent for minors has been corrected to 10 years, to make it consistent with the text of the protocol.

In addition, Dr. Lim Pharath has been replaced by Mr. Phektra Chim, who will be responsible for the in vitro drug resistance assays.

In response to review by the NAMRU-2 IRB 22JUN09 the following changes were made:

1.The following material was added to the background to provide additional information on the efficacy and tolerability of piperaquine-dihydroartemisinin:

“Piperaquine-dihydroartemisinin is recommended by the World Health Organization for containment of drug resistant *P. falciparum* malaria in Cambodia and is now the first-line treatment recommended by the Cambodian Ministry of Health for uncomplicated *P. falciparum* in the Phase 1 containment zone. The efficacy and tolerability of piperaquine-dihydroartemisin has recently been reviewed (4). In brief, in 14 studies involving 2636 patients with uncomplicated *P. falciparum* malaria, both adults and children, cure rates at 28 days were 97-98%. The therapy was well tolerated; common adverse events occurring in 1-10% of subjects included nausea, vomiting, anorexia, headache, dizziness, diarrhea, and abdominal pain, all of which are also symptoms of malaria. There were no serious adverse events reported.”

2. The section “Justification for the use of human subjects” was renamed “Justification for the use of human subjects, including children” and the following material added:

“In areas where malaria transmission occurs adults may have partial clinical immunity to malaria. Even drug resistant malaria parasites may be cleared by treatment with drugs to which the parasite is resistant, because of the synergistic effect of partial immunity and partially effective drugs. Efficacy studies which exclude children may therefore overestimate efficacy and do not provide efficacy data for the population at greatest risk of death from malaria. It is therefore important to measure efficacy in a population which includes children.”

New in version 1_2, 03 October 2009:

In order to bring the treatment schedules in the protocol in line with revised Cambodian Ministry of Health guidelines and WHO recommendations for treatment with mefloquine-artesunate and piperaquine-DHA, the following paragraphs,

*“i. Mefloquine-artesunate*

Each subject will be treated with 12 mg/kg of artesunate over 3 days and 25 mg/kg of mefloquine on Day 0, divided into two doses, one in the morning and the other in the afternoon (or 8 hours apart). Dosage will be based on weight up to a maximum dose of 1250 mg mefloquine/600 mg artesunate.

*ii. Piperaquine-DHA*

One tablet of piperaquine-DHA contains 320 mg piperaquine and 40 mg of DHA. An adult dose consisted of four doses of two tablets, given at 0, 8, 24 and 48 h. The approximate total adult dose is 48/6 mg/kg (piperaquine/DHA). For children under 50 kg, weight-based dosing will be used;12.8/1.6 mg/kg piperaquine/DHA given at the same time intervals as for adults. “

have been replaced with the following modified versions:

**i. Mefloquine-artesunate**

**Each subject will be treated with 12 mg/kg of artesunate and 25 mg/kg of mefloquine over 3 days, divided as shown in the following table. Dosage will be based on weight up to a maximum dose of 1250 mg mefloquine/600 mg artesunate**.

| **Weight (kg)** | **Age (years)** | **Day 0** | **Day 1** | **Day 2** |
| --- | --- | --- | --- | --- |
| **10-15** | **5-6** | **1A** | **1M+1A** | **1A** |
| **16-25** | **6-11** | **1M+2A** | **1M+2A** | **2A** |
| **25-35** | **11-15** | **1M+3A** | **1M+3A** | **1M+3A** |
| **≥35** | **≥15** | **2M+4A** | **2M+4A** | **1M+4A** |

**M-mefloquine 250 mg tablet;A-artesunate 50 mg tablet. Table from “National Treatment Guideline for Malaria in the Kingdom of Cambodia.”**

**ii. Piperaquine-DHA**

**One tablet of piperaquine-DHA contains 320 mg piperaquine and 40 mg of DHA. An adult dose consists of three doses of three tablets, given as one dose per day on Day 0, Day 1, and Day 2. For children under 50 kg, weight-based dosing will be used, as shown in the fo**llowing table.

| **Weight (kg)** | **Age (years)** | **Day 0 (tablets)** | **Day 1 (tablets)** | **Day 2 (tablets)** |
| --- | --- | --- | --- | --- |
| **10-19** | **5** | **1** | **1** | **1** |
| **19-30** | **5-10** | **1 ½** | **1 ½** | **1 ½** |
| **30-40** | **10-15** | **2** | **2** | **2** |
| **>40** | **>15** | **3** | **3** | **3** |

**Table from “National Treatment Guideline for Malaria in the Kingdom of Cambodia.”**

The following minor changes were made in version 1.3 on 10 February 2011:

1. ICF, the contact person for questions concerning subject rights was changed from NAMRU-2 IRB Chair to CDR David Fryauff, NMRC IRB Chair.

2. The following investigators were deleted from the protocol; LT Michael Kinzer, Ika Sutanti, and Frederic Ariey.

3. The previous NAMRU-2 Commanding Officer, CAPT Trevor Jones, has been replaced by CAPT Gail Hathaway.

4. The ending date of the protocol has been extended by one year to 31 October 2012

5. In section C.2, Multicenter Organizational Plan for IRB Review and Approval, the following sentence has been added “Continuing review of the study for NAMRU-2 will be conducted by the IRB of the Naval Medical Research Center, Silver Spring Maryland, pending re-establishment of the NAMRU-2 IRB in Phnom Penh.”

The following minor changes were made in version 1.4 on 22 March 2011:

# 1. Reference to child assent was removed from the protocol (Section B.1.c) and the informed consent document. Waiver of assent on the part of minor participants was requested on the grounds that (a) the study offers an intended benefit to the minor child, (b) that benefit is important, and (c) the benefit is not available outside the context of the study. Specifically, the risk of drug resistant *P. falciparum* malaria is very real in Cambodia; there is a small, but significant (5-10%) chance that treatment with current, standard regimens will fail. The 6 week follow-up which is part of the study protocol makes early detection and treatment of recurrent parasitemia more likely, significantly reducing the risk of severe morbidity or mortality from treatment failure. Although the same standard treatment regimens used in this study are available outside the context of the study, the careful six week follow-up is not available outside of the study.

# a. In Section B.1.c, the sentence “Assent will be obtained from all children greater than 10 years of age” was deleted and replaced with the following text:

“Assent will not be obtained from minor children, because the study offers an important benefit to study participants which is not available outside the context of the study. Specifically, the risk of drug resistant *P. falciparum* malaria is very real in Cambodia; there is a small, but significant (5-10%) chance that treatment with current, standard regimens will fail. The 6 week follow-up which is part of the study protocol makes early detection and treatment of recurrent parasitemia more likely, significantly reducing the risk of severe morbidity or mortality from treatment failure. Although the same standard treatment regimens used in this study are available outside the context of the study, the careful six week follow-up is not available outside of the study. “

b. In the informed consent document, the signature line for child assent was deleted.

# PART III: SIGNATURES AND INVESTIGATOR ASSURANCE AGREEMENT

**A. SIGNATURES**

Signatures below acknowledge that this research proposal has been reviewed and that the investigator agrees with the proposal as submitted.

___________________________________________________________________________

Lek Dysoley Date

Principal Investigator

___________________________________________________________________________

William O. Rogers Date

Co-Investigator

___________________________________________________________________________

Duong Socheat Date

Co-Investigator

___________________________________________________________________________

Sem Rithy Date

Co-Investigator

___________________________________________________________________________

Phektra Chim Date

Co-Investigator

___________________________________________________________________________

Sinuon Muth Date

Medical Monitor

B. INVESTIGATOR ASSURANCE AGREEMENT

For all NAMRU-2 investigators.

**INVESTIGATOR ASSURANCE AGREEMENT**

I, a research investigator, promise to protect the ethical rights and welfare of human participants enrolled in a research protocol entitled, “Prevalence of Genetic Mutations Associated with Drug Resistance in *P. falciparum* in Cambodia.” I understand and accept my responsibility for the protection of human research subjects as found in The Belmont Report and the provisions of Title 32 Code of Federal Regulations Part 219 (Protection of Human Subjects), Department of Defense (DoD) Directive 3216.2 (Protection of Human Subjects in DoD-Supported Research), Secretary of the Navy Instruction (SECNAVINST) 3900.39D (Protection of Human Subjects), , Naval Medical Research Center Instruction (NAVMEDRSCHCENINST) 3900.6D (Protection of Human Subjects In Medical Research), and all other relevant regulations concerning standards of conduct for the Department of Defense and the Department of the Navy. I will abide by all applicable laws and regulations relevant to the ethical protection of the rights and welfare of human research subjects; and I guarantee that I will follow the most restrictive regulation in all cases and without exception. In the event any question regarding my obligations arises during the conduct of this project, I will consult with the Institutional Review Board Chair and any other human research authorities in my chain of command.

Signatures and dates: (DD/MM/YY)

____________________________ ___/__/__

William O. Rogers

Navy Lead Investigator

### COMMANDING OFFICER SIGNATURE PAGE

____________________________________ ____________

G.L. Hathaway DATE

CAPT, MSC, USN

Commanding Officer

U.S. Naval Medical Research Unit 2 (US NAMRU-2)

Pearl Harbor, Hawaii

The Commanding Officer’s signature indicates that the Principal Investigator and co-investigators have obtained all necessary approvals (IRBs; US NAMRU-2 and Cambodian Ministry of Health) and permission to initiate the work. This signature must be present prior to commencement of this work. Commanding Officer endorsement of IRB recommendations are distinct and do not substitute as an endorsement to initiate the work outlined in this protocol.

# PART IV: PROTOCOL CRITICAL ELEMENTS

## A. SCIENTIFIC BACKGROUND AND OBJECTIVES

### 1. Background

The spread of drug resistant *P. falciparum* has complicated efforts to control malaria, and can lead to unnecessary mortality if ineffective drugs remain the standard of care after drug-resistant strains become established (12,17). In Southeast Asia, resistance to multiple anti-malarial drugs, including chloroquine, sulfadoxine-pyrimethamine, quinine, and mefloquine is common (5). In the face of this situation, countries in the region have adopted artemisinin combination therapies (ACT) as first-line treatment for uncomplicated *P. falciparum* malaria. In Cambodia, current Ministry of Health guidelines recommend mefloquine-artesunate as first-line therapy for uncomplicated *P. falciparum* malaria. Recently, however, there has been growing evidence of resistance to mefloquine-artesunate at several sites in Cambodia and in Thai provinces bordering Cambodia. In 2003, a regimen of 25 mg/kg mefloquine in two divided doses on day 0, and 12 mg/kg oral artesunate divided into two doses on day 0 and day 1, in Trat, Thailand, along the Cambodian border, produced an adequate clinical and parasitological response (ACPR) at day 28 in only 78.6% of 44 patients with uncomplicated *P. falciparum* malaria; at three other sites in Thailand the same regimen had an efficacy of >90% (13). In 2002 in Pailin, a Cambodian province bordering Trat, a similar regimen produced ACPR at 28 days in 85.7% of subjects (2); in this study almost one third of patients received less than 12 mg/kg artesunate and 20 mg/kg mefloquine, because blister pack doses were administered according to age rather than to weight. Nonetheless, the ACPR was >90% at sites outside of Pailin where the same dosing scheme was used. In 2004, using dosing based on weight rather than age, the 42 day ACPR was 79.3% in Pailin (2). More recently, in 2006-2007 in Chumkiri, a site in southern Cambodia, far from the Thai border, we found that a regimen of 12 mg/kg artesunate over three days and 25 mg/kg mefloquine on day 0 gave ACPR at 42 days in only 81.2% of subjects (10). Both the Thai and Cambodian studies used directly observed therapy, and the Cambodian studies also used PCR correction for re-infection (16). These findings suggest that resistance to artesunate-mefloquine may be emerging at the Thai-Cambodian border and may have begun spread beyond the immediate border area.

The Cambodian Ministry of Health has taken steps to contain the possible spread of *P. falciparum* resistant to mefloquine-artesunate. In collaboration with the World Health Organization and the Gates Foundation, the Ministry of Health designed a program to confirm the presence of artemisinin resistance, to contain its spread, and to eliminate it before it can spread further. This plan defines Phase 1 and Phase 2 containment and elimination zones (Figure 1), including the areas in which significant failure of mefloquine-artesunate has already been documented (Phase 1) and areas at risk because they are adjacent to Phase 1 (Phase 2). In the Phase 1 containment area, mefloquine-artesunate has been replaced by piperaquine-dihydroartemisinin as first-line therapy for uncomplicated *P. falciparum* malaria. Piperaquine-dihydroartemisinin is recommended by the World Health Organization for containment of drug resistant *P. falciparum* malaria in Cambodia and is now the first-line treatment recommended by the Cambodian Ministry of Health for uncomplicated *P. falciparum* in the Phase 1 containment zone.The efficacy and tolerability of piperaquine-dihydroartemisin has recently been reviewed (4). In brief, in 14 studies involving 2636 patients with uncomplicated *P. falciparum* malaria, both adults and children, cure rates at 28 days were 97-98%. The therapy was well tolerated; common adverse events occurring in 1-10% of subjects included nausea, vomiting, anorexia, headache, dizziness, diarrhea, and abdominal pain, all of which are also symptoms of malaria. There were no serious adverse events reported. In addition, the Ministry of Health will conduct a large scale screening and treatment campaign in Phase 1 using PCR for diagnosis of inapparent infections and treatment with Malarone (proquanil-atovaquone). It is anticipated that similar tactics will be used in the Phase 2 containment zone in following years.

The success of this containment program may depend on the extent to which mefloquine-artesunate resistance has already spread beyond the Phase 1 containment zone, and on the continuing efficacy of piperaquine-DHA and Malarone in treating uncomplicated *P. falciparum* in the Phase 2 containment zone. In previous studies, we identified sites at risk for mefloquine-artesunate resistance by screening for the prevalence of amplified *pfmdr1* genes. Amplifications at this locus are strongly associated with in vivo resistance to mefloquine (14). We recently screened approximately 700 parasite samples from cases of uncomplicated *P. falciparum* malaria from 5 provinces within and outside the containment zones. The highest average *pfmdr1* copy number (~2.5) was found, unsurprisingly, at Chumkiri, the site in southern Cambodia where we identified significant resistance to mefloquine-artesunate (10). Sites in eastern Cambodia, outside both containment zones had average *pfmdr1* copy numbers <1.5. A site in containment zone 2, Trapeang Prasat, Oddarmeanchey Province, had an elevated mean *pfmdr1* copy number (~2), suggesting a risk of mefloquine-artesunate resistance. We therefore plan to assess the in vivo efficacy of mefloquine-artesunate, piperaquine-DHA, and Malarone at this site. We will conduct an in vivo efficacy study following guidelines recommended by WHO and the World Anti-malarial Resistance Network (WARN) (8,16). Subjects will be randomized to receive one of the three standard therapies, and microscopists and laboratory technicians will not be informed as to which treatment each subject has received; there will, however, be no more formal blinding of the study. We will also perform in vitro drug resistance assays on parasite samples collected during the study, and will screen the samples for previously identified molecular markers of drug resistance.

### 2. Objectives and Hypotheses

Primary Objective: To measure the proportion of uncomplicated *P. falciparum* malaria patients who have an adequate clinical and parasitological response following treatment with mefloquine- artesunate, piperaquine-DHA, or Malarone. It is important to note that the objective is to measure the efficacy of each individual regimen, rather than to compare the efficacies of each regimen, one to another.

Secondary Objective: To determine whether previously identified molecular markers of drug resistance, and in vitro drug resistance assays, predict clinical outcome for the three treatment regimens under study.

Primary Hypothesis: The upper bound of the 95% confidence interval for the proportion of ACPR in subjects treated with mefloquine-artesunate will be <0.9. The lower bound of the 95% confidence interval for the proportion of ACPR in subjects treated with piperaquine-DHA or Malarone will be >0.9.

Secondary Hypothesis: The odds ratio for treatment failure will be >2 in the following cases (a) patients treated with mefloquine artesunate whose Day 0 parasites have pfmdr1 copy number >1.5, (2) patients treated with Malarone whose Day 0 parasites have either *pfdhfr* S108N + N51I , or *pfcytb* Y268N. Mean IC50 values for treatment drugs will be statistically significantly higher in subjects who do not have ACPR than in those who do.

## B. EXPERIMENTAL METHODS

### 1. Experimental procedures and rationale

#### a. Justification for the use of human subjects, including children

The objective of the study is to measure the clinical effectiveness of commonly used anti-malarial drugs against *P. falciparum* malaria. It is possible to conduct in vitro drug resistance assays, however the clinical cure rate is not accurately predicted by the in vitro drug resistance assays, particularly in settings in which there is some degree of acquired immunity to malaria. Therefore, information about clinical cure rates needed to make decisions about anti-malarial drug policy can only be obtained from human studies. In areas where malaria transmission occurs adults may have partial clinical immunity to malaria. Even drug resistant malaria parasites may be cleared by treatment with drugs to which the parasite is resistant, because of the synergistic effect of partial immunity and partially effective drugs. Efficacy studies which exclude children may therefore overestimate efficacy and do not provide efficacy data for the population at greatest risk of death from malaria. It is therefore important to measure efficacy in a population which includes children.

#### b. Study Location

The study will be conducted at the Trapeang Prasat Health Center in Oddarmeanchey Province, Cambodia. This site is in a rural, agricultural area about 8 hours by road from Phnom Penh. Falciparum malaria is common in the area around the health center. The health center participates in a current surveillance program to measure the prevalence of molecular markers of drug resistance in *P. falcparum*. In the first six months of surveillance, the health center enrolled 450 subjects over age 5. Of these, 433 (96%) had parasitemia > 1000 parasites/µl. The mean pfmdr1 copy number was 1.89 (95% CI 1.78, 2.00) and the proportion of samples with copy number >1.5 was 0.49. Although this prevalence of amplified *pfmdr1* genes is lower than at Chumkiri, the site at which we found an unexpectedly high failure rate for mefloquine-artesunate (10), it is elevated compared to sites in eastern Cambodia from which mefloquine resistance has not been reported. We expect, therefore, both that the number of eligible subjects will be sufficient and that there is a reasonable likelihood of finding mefloquine artesunate resistance in this site.

All laboratory analyses will be conducted at one of the collaborating Cambodian institutions, the National Malaria Center, or the Institute Pasteur de Cambodge. Confirmation of microscopic diagnosis of malaria will be performed at the National Malaria Center. RT-PCR based SNP and gene amplification assays for detection of molecular markers of drug resistance will be performed at Institute Pasteur or CNM; both these laboratories have experience in performing the RT-PCR assays. In vitro drug resistance assays will be performed at the Pasteur Institute.

#### c. Volunteer recruitment and enrolment

A study investigator at the clinic will ask adult and child patients entering the clinic and found to have a qualifying blood film examination if they are willing to consider volunteering for this study. The study investigator will explain the study to those interested in volunteering. He/she will review the informed consent document with the potential participant or the parent or guardian of eligible minors. Consent will be obtained from either the study participant, if a legal adult, or from the parent or guardian if the subject is a minor. In Cambodia the legal age of majority is 18 years. Assent will not be obtained from minor children, because the study offers an important benefit to study participants which is not available outside the context of the study. Specifically, the risk of drug resistant *P. falciparum* malaria is very real in Cambodia; there is a small, but significant (5-10%) chance that treatment with current, standard regimens will fail. The 6 week follow-up which is part of the study protocol makes early detection and treatment of recurrent parasitemia more likely, significantly reducing the risk of severe morbidity or mortality from treatment failure. Although the same standard treatment regimens used in this study are available outside the context of the study, the careful six week follow-up is not available outside of the study.

The study investigator will evaluate the detailed inclusion/exclusion criteria listed below to determine participant eligibility. Otherwise eligible women of childbearing age (≥12 years of age) will be asked to provide a urine sample for pregnancy testing and will be considered eligible only if the test is negative. Eligible, consenting subjects will be enrolled; ineligible persons will be referred for routine therapy in the clinic.

#### d. Inclusion and exclusion criteria

The inclusion criteria are as follows:

1) Age≥5 years

2) *P. falciparum* parasitemia >1000/µl blood, but <100,000/µl

3) Axillary temperature > 37.5oC or rectal or tympanic temperature > 38.0^o^C, or history of fever within past 24 hours.

4) Negative urine pregnancy test

5) Ability to swallow oral medication

6) Availability for follow-up over 42 days

7) Freely provides informed consent

Specific exclusion criteria are the following:

1) Age < 5 years

**Justification: Phlebotomy is more difficult and not well accepted for young children in Cambodia.**

2) Mixed infection (e.g. *P. falciparum* and *P. vivax*)

**Justification: Inclusion of subjects with mixed infections would complicate the analysis and prevent straightforward measurement of treatment efficacy for *P. falciparum* and *P. vivax*.**

3) Positive urine HCG test for pregnancy

**Justification: One of the treatment drugs, Malarone, is not recommended in pregnancy.**

4) History of epilepsy or psychiatric illness

**Justification: One of the treatment drugs, mefloquine, is contraindicated in patients with a history of epilepsy or psychiatric illness.**

5) Any of the following WHO criteria for severe malaria (17)

Clinical malaria with any one or more of the clinical manifestations listed below

i) Cerebral Malaria. Unrousable coma or Blantyre Coma Score of 3 or less; coma persists for more than 30 minutes after fits have ceased and normal CSF findings.

ii) Repeated or prolonged generalised convulsions. Generalised convulsions lasting more than 30 minutes or more than 2 fits in 24 hours despite cooling

iii) Severe anaemia

Haemoglobin < 5g/dL

iv) Respiratory Distress

Presence of any of the following; alar flaring, chest recession (intercostals or sub costal), use of accessory muscles of respiration; abnormally deep (acidotic) breathing.

v) Hypoglycaemia (blood glucose < 2.2mM/L[<40 mg/dL])

vi) Circulatory collapse (systolic bp <50mm Hg)

vii) Renal failure (urine output less than 12ml/kg/24hrs; or serum creatinine conc >3.0mg/dL)

viii) Malarial haemoglobinuria

ix) Other manifestations

hyper parasitaemia (>100,000 /µl)

Jaundice (serum bilirubin ≥ 3.0mg/dL)

Impaired consciousness (but rousable)

**Justification: The treatment regimens evaluated in this project are not appropriate for treatment of severe malaria. Use of such oral regimens in severe malaria would place the volunteer at unnecessary risk.**

6) Serious co-morbid conditions requiring hospitalization (including, but not limited to severe renal or liver disease, uncontrolled diabetes, systemic bacterial infections).

**Justification: Such severe disease may reasonably be expected to alter the pharmacokinetics of the anti-malarial drugs and therefore to complicate the interpretation of results.**

7) On-going antibiotic therapy

**Justification: Many antibiotics used to treat bacterial infections have greater or lesser activity against *Plasmodium*. Their use would confound the interpretation of results.**

8) History of hypersensitivity to any of the study drugs

**Justification: Such a history would be a contraindication to the use of the specified anti-malarials.**

9) Plans to leave area during next 42 days or to be unavailable for scheduled follow-up

**Justification: Unavailability for follow-up would make assessment of treatment success or failure impossible.**

10) Nursing mother

**Justification: Malarone is not recommended for nursing mothers.**

11) Any other condition which, in the judgment of the study physician would make participation in the study unsafe for the potential volunteer.

**Justification: The study team has an obligation to protect the safety of potential volunteers.**

#### e. Anti-malarial therapy

This is a randomized, open-label trial of the efficacy of three standard antimalarial regimens. Subjects who agree to participate in the study will be randomized to the different, standard anti-malarial treatments as follows. Three hundred sequentially numbered envelopes will be prepared. A statistician, otherwise unconnected with the study, will randomly distribute cards indicating one of the three regimens into each envelope. The envelopes will be sealed and signed across the seal. When a subject has enrolled in the study and provided informed consent, the sequential envelope corresponding to the subject’s study number will be opened, and the subject will be given the treatment regimen indicated on the enclosed card. The drug doses in each regimen will be as follows:

*i. Mefloquine-artesunate*

Each subject will be treated with 12 mg/kg of artesunate and 25 mg/kg of mefloquine over 3 days, divided as shown in the following table. Dosage will be based on weight up to a maximum dose of 1250 mg mefloquine/600 mg artesunate.

| Weight (kg) | Age (years) | Day 0 | Day 1 | Day 2 |
| --- | --- | --- | --- | --- |
| 10-15 | 5-6 | 1A | 1M+1A | 1A |
| 16-25 | 6-11 | 1M+2A | 1M+2A | 2A |
| 25-35 | 11-15 | 1M+3A | 1M+3A | 1M+3A |
| ≥35 | ≥15 | 2M+4A | 2M+4A | 1M+4A |

M-mefloquine 250 mg tablet;A-artesunate 50 mg tablet. Table from “National Treatment Guideline for Malaria in the Kingdom of Cambodia.”

*ii. Piperaquine-DHA*

One tablet of piperaquine-DHA contains 320 mg piperaquine and 40 mg of DHA. An adult dose consists of three doses of three tablets, given as one dose per day on Day 0, Day 1, and Day 2. For children under 50 kg, weight-based dosing will be used, as shown in the following table:

| Weight (kg) | Age (years) | Day 0 (tablets) | Day 1 (tablets) | Day 2 (tablets) |
| --- | --- | --- | --- | --- |
| 10-19 | 5 | 1 | 1 | 1 |
| 19-30 | 5-10 | 1 ½ | 1 ½ | 1 ½ |
| 30-40 | 10-15 | 2 | 2 | 2 |
| >40 | >15 | 3 | 3 | 3 |

Table from “National Treatment Guideline for Malaria in the Kingdom of Cambodia.”

*iii. Malarone*

One adult tablet of Malarone contains 250 mg of atovaquone and 100 mg of proguanil hydrochloride. For adults weighing >40 kg the daily dose is 4 tablets; for subjects under 40 kg the dosage is as follows: 11‐20 kg, 1 tablet; 21‐30 kg, 2 tablets; 31‐40 kg, 3 tablets. The daily dose is given for three days, day 0, day 1, and day 2.

The doses for all medications will be given orally with water. Patients will remain in the clinic for observation for one hour after each dose. If they vomit within the hour, the dose will be repeated. If they vomit within an hour of the repeated dose, they will be withdrawn from the study and referred to a local clinician for possible parenteral malarial treatment.

*iv. Rescue therapy*

Treatment failures, defined as persistent or recurrent parasitemia during the 42-day follow-up, will be treated with oral quinine sulfate 10 mg salt/kg tid x 7 days plus tetracycline 1 gm. x 7 days.

*v. Adjunctive therapy*

Adjunctive therapy, including analgesics, anti-pyretics, or rehydration, will be given at the discretion of the health center medical staff, and is not considered part of the study treatment.

f. Source of Study Drugs

All study drugs will be provided by the Cambodian Ministry of Health, which procures them from WHO-recommended producers who follow current Good Manufacturing Practices.

**g. Follow-up**

Subjects are asked to re-visit the study unit on days 1, 2, 3, 7, 14, 18, 21, 28, 35 and 42, plus any other day that he/she feels sick, in order to monitor clinical recovery/recurrence of malaria symptoms. In the event that a subject fails to re-visit the study unit on schedule, a member of the study team will visit the subject and encourage the subject to visit the clinic for the scheduled follow-up. Any adverse events associated with the treatment regimen will be recorded.

Any time a subject is referred to the physician for a reason other than routine follow-up, the physician-subject contact will be recorded.

The following conditions constitute cause for removing a study subject from the study:

1. An expressed desire by the subject to terminate participation

2. A determination by the on-site physician that the subject risks his/her health by further participation

3. Development of a serious disease, including severe malaria (WHO criteria, Appendix VII), that requires referral to hospitalized treatment

4. Development of an infection requiring antibiotic therapy

5. Inability to locate a subject on two consecutive scheduled visits

6. Ingestion of any anti-malarial by subject outside protocol parameters

The following prompts administration of rescue therapy:

1. Asexual parasitemia on day 2 > day 0

2. Asexual parasitemia fails to clear by day 4

3. Asexual parasitemia recurs on or between days 4 and 42.

#### f. Laboratory procedures

##### Malaria thick and thin blood films

Thick and thin blood smears will be stained with Giemsa and examined by a certified microscopist using 1000X oil immersion light microscopy. At least 200 ocular fields will be examined before a negative diagnosis is rendered. The microscopist records the number of asexual and sexual forms (separately) per 200 white blood cells in the thick smear. This is later converted to parasites/µl with a conversion multiple of 40 (assumes 8000WBC/µl) for analysis. All smears will be reviewed by a second certified microscopist at CNM. Slides for which results are discrepant will be reviewed by a third certified microscopist and the majority reading considered definitive. In the event that more than 10% of the reviewed slides show discrepancies, all study slides will be reviewed before data analysis.

##### Hematocrit determination

Hematocrit will be measured by capillary tube centrifugation.

##### In vitro resistance assays

We will assess the *in vitro* drug sensitivity of the *P. falciparum* isolates by use of a classical isotopic 48-hour test [15]. In brief, we will obtain anti-malarial drugs from IMTSSA (Institut de Medicine Tropical, Service de santé des armées, Marseille, France). We will prepare stock solutions of the antimalarials in methanol and further two-fold serial dilutions in distilled water (Biosedra, France). We will coat two wells of a Falcon 96-well, flat-bottom plate (ATGC, France) with each drug concentration, dry the plates in a sterile cabinet, and store them at 4°C until use. We will monitor their suitability for *in vitro* testing using reference strains of *P. falciparum* with known drug sensitivities. We will use only blood samples with a parasitemia of at least 40,000 parasites/µl. We will wash fresh blood samples three times with RPMI 1640 medium (GibcoTM, Invitrogen Corporation, France) by centrifugation (800g, 10 min, 4oC) and then test them directly without culture adaptation. We will suspend the infected erythrocytes (1.5% haematocrit, 0.1% - 1% parasitaemia) in complete RPMI medium supplemented with 10% of decomplemented human AB+ serum (Biomedia, France), buffered with 25 mM/l Hepes, 11mM/l D-(+)-glucose 25 mM/l NaHCO3, and containing [8-3H] hypoxanthine (0.5 µci/well; Amersham Biosciences, France), and distribute the mixture (200 µl per well) into the 96-well test plates pre-coated with anti-malarial drugs. Each plate will include two drug-free control wells and one control well without parasites. We will incubate the plates for 48h at 37°C in a 5% CO2 atmosphere and then lysed the cells by freeze-thawing. After collection on glass-fiber filter paper using a cell harvester, we determined the amount of [3H] hypoxanthine incorporated into the parasites nucleoprotein using a Wallac MicroBeta Trilux counter (Perkin Elmer, France). We used a log probit approximation to determine the 50% inhibitory concentration (IC50), defined as the concentration at which 50% of the incorporation of [3H] hypoxanthine was inhibited, as compared with the drug-free control wells.

The in vitro resistance assay requires a starting parasitemia of at least 0.1%. Therefore, it will not be performed on samples from subjects with a starting parasitemia of <40,000 parasites/µl (160parasites/200 wbc).

##### Parasite genotyping studies

Filter paper blood spots will be collected on #1 Whatman Filter Paper and stored individually in zip-lock plastic bags with desiccant. Dried blood blots will be cut into small pieces, placed in 1.5 ml micro centrifuge tube and lysed in 1 ml of sterile water for 10 minutes at room temperature. During this step, the tubes will be vortexed every 1-2 minutes. The tubes will then be centrifuged at 15,000 rpm for 5 minutes. After pouring off the supernatant, DNA precipitate will be resuspended in 10 volume of 5% chelex-100 (Biorad Laboratories Inc. Hercules, CA), and incubated for 20 minutes at 56^o^C with a brief vortex at high speed before and after incubation. The tubes will be placed in 100^o^C heating block for 8 minutes and vortexed briefly at high speed after incubation. After a final centrifugation at 15,000 rpm for 2 minutes to precipitate chelex-bound organic material, DNA-containing supernatants will be removed by pipette for PCR amplification. Yields from blood spots of about 2 cm diameters may be expected to be in the range of 50 to 100 ng total DNA. If alternative or automated extraction methods become available during the course of the study, they may be used.

Analysis of amplification of the Pfmdr gene will be conducted as follows(7,15). Multiplex PCR will be used to amplify products from both the pfmdr1 and β-tubulin genes in a single tube. PCR will be performed in a total volume of 25 µl containing PCR buffer, 3.0 mM MgCl2, 300 µM of each deoxynucleoside triphosphate, and Taq DNA polymerase, 300 nM each Pfmdr1 primer, 100 nM of each β-tubulin primer, 150 nM of Pfmdr probe labeled with 5’ Fam and 3’ Tamra and 100 nM of β-tubulin probe labeled with 5’ Vic and 3’ Tamra. Each assay will be performed in duplicate. Reactions will be carried out in a Rotorgene Real Time PCR system using the following cycling conditions: 1x 50^o^C for 2 min, 1x 95^o^C for 15 min and 50 cycles of 95^o^C for 15 sec and 60^o^C for 1 minute. Results will be were accepted as valid only if the copy number for control DNA samples are 0.8-1.2 for 3D7 and 2.8-3.2 for W2Mef , and if the difference between duplicate copy numbers is <50% of the average. The efficiency, E, will be calculated from the slope of a standard curve made from known dilutions of a reference DNA (E=10^-1^/slope). The copy number will then be calculated as: Copy# = EbtCtbt/EmdrCtmdr. Where Ebt and Emdr are the efficiencies for β-tubulin and Pfmdr, respectively, and Ctbt and Ctmdr are the corresponding number of cycles to reach threshold.

SNPs within the Pfmdr gene will be detected using RT-PCR as follows(6,9). The four polymorphic sites in pfmdr1 to be analyzed are Asn86/Tyr86, Tyr184 /Phe184, Ser1034/Cys1034, and Asn1042/Asp1042. Briefly, fluorescent-labeled minor groove binding (MGB) probes have been designed specific to the polymorphic site for wild-type or mutant species. Following amplification, the genotype of the four polymorphic sites was determined by increase in fluorescent emission of the wild-type or mutant probes. The relevant primers and probes are shown in the following table:

| Pf MDR 1 Point mutation | Primer/Probe | Oligonucleotide Sequence (5’ 🡪 3’) |
| --- | --- | --- |
| 86 | Forward | ATGTGCTGTATTATCAGGAGGAAC |
|  | Reverse | TTGTACTAAACCTATAGATACTAATGATAATATTATAGG |
|  | Asn (wt*) probe | 6FAM-ACCTAAAT**T**CATGTTCTTT-MGB-NFQ |
|  | Tyr (mut**) probe | VIC-ACCTAAAT**A**CATGTTCTTT-MGB-NFQ |
|  |  |  |
| 184 | Forward | AAGATGGACAATTTCATGATAATAATCCT |
|  | Reverse | AATACATAAAGTCAAACGTGCATTTTTTA |
|  | Tyr (wt) probe | 6FAM-CTTTTTAGGTTTAT**A**TATTTGGT-MGB-NFQ |
|  | Phe (mut) probe | VIC-CTTTTTAGGTTTAT**T**TATTTGGT-MGB-NFQ |
|  |  |  |
| 1034 | Forward | AAAAAGAAGAATTATTGTAAATGCAGCTT |
|  | Reverse | GGATCCAAACCAATAGGCAAAA |
|  | Ser (wt) probe | 6FAM-ATTC**A**GTCAAAGCGCT-MGB-NFQ |
|  | Cys (mut) probe | VIC-ATTC**T**GTCAAAGCGCT-MGB-NFQ |
|  |  |  |
| 1042 | Forward | AAAAAGAAGAATTATTGTAAATGCAGCTT |
|  | Reverse | TTTCCAGCATAACTACCAGTAAATATAAAAG |
|  | Asn (wt) probe | 6FAM-CAATTATTTATT**A**ATAGTTTTGC-MGB-NFQ |
|  | Asp (mut) probe | VIC-AATTATTTATT**G**ATAGTTTTGC |
|  |  |  |

Analysis of mutations in other genes associated with malaria drug resistance will be performed by RT-PCR, PCR-RFLP analysis or allele specific amplification(3,11). For example, the presence of the K76T mutation in the Pfcrt gene associated with chloroquine resistance will be detected by nested PCR amplification of a 145 bp fragment including the mutation site. Digestion with the restriction enzyme *Apo*I distinguishes between the sensitive and resistant alleles.

Multiplexed analysis of SNPs associated with drug resistance will be performed using Luminex technology as follows, in a modification of previously described techniques(1). The Luminex allele-specific primer extension product (ASPE) uses gene specific primers that are placed 5’ upstream of the variable base (SNP) in the amplified sequence with a variable 3’ end nucleotide that will be used to define the allele or SNP. These primers will be used to generate a fluorescently labeled allele-specific primer extension product (ASEP) using the PCR amplified gene product, DNA polymerase, SNP specific biotin labeled ddNTP and each of the other three non-labeled ddNTPs. The SNP specific primers contain a unique identifying sequence that is complimentary to a c-sequence covalently attached to fluorescent microspheres. Following reaction, the newly generated ASEP will be captured by the c-sequence labeled microsphere and analyzed by the Luminex 100 flow cytometer after development with streptavidin-phycoerythrin (SA-PE). Each microsphere will be assayed for its characteristic fluorescence and SA-PE. Only microspheres that contain both fluorescent tags will be considered a positive result. By knowing which biotin-labeled ddNTP was present in the well and c-sequence each microsphere possesses, the sequence of the variable 3’ end nucleotide can be determined.

### 2. Sample Size

The sample size is based entirely on the primary objective and primary hypotheses. Several approaches to calculating the sample size yield a sample size of ~100 subjects per group. For example, consider that we want to demonstrate that mefloquine-artesunate has an efficacy of less than 90%. If the observed efficacy is 80%, as observed in our recent trial in Kampot Province, then for a sample size of 100, the upper 95% confidence interval for the efficacy is 87%. Consider that we want to show that the efficacy of Malarone or piperaquine-DHA is greater than 90%. For a sample size of 100, if the observed efficacy of either regimen is ≥97.5%, as observed in a previous WHO study in Oddarmeanchey, then the lower 95% confidence interval will be at 91%. Similarly, in order to have a power of 90% to detect the difference between an efficacy of 95% in one regimen and 80% in another, a sample size of 100 is required.

### 3. Data Analysis

Data will be recorded on standardized case report forms, entered into an Access database, and exported for analysis in STATA, SPSS, and Mathematica.

Therapeutic Efficacy. Drug efficacy will be calculated as the percent of subjects not requiring alternative therapy during the 42-day course of the test and reported with 95% confidence intervals. This will be the primary analysis and, as recommended by WHO will be conducted as a per protocol (rather than intent to treat) analysis. One disadvantage of this approach is that data from subjects who drop out or are lost to follow up during the follow-up evaluation period are not used. As a secondary, alternative approach we will calculate the failure rate using Kaplan-Meier survival analysis. The failure rate will be calculated as 1-Π(ri-di)/ri , where ri is the number of subjects without a recrudescence just before time ti, di is the number who had a recrudescence at time ti, and Π is the product of the fractions, (r-d)/r, at all points, i. The drawback to this analysis is that it makes the assumption that the risk of recrudescence in the patients lost to follow-up is the same as that in those not lost. This may not be a good assumption. Therefore, sensitivity analyses will be performed to determine the effect on the reported failure rate of the extreme assumptions that either all patients lost to follow-up had recrudescences, or that they all did not have them.

Predictors of treatment failure. Data will be analyzed using standard statistical packages such as Stata, SAS or SPSS. Subjects will be categorized based on therapeutic outcomes, presence of the various genetic markers and other demographic and parasite factors that are putative determinants of treatment failure, e.g. age, parasite density, parasite clearance time. Bivariate associations will be examined between these markers and the outcomes. Chi square or Fisher’s exact tests for two-tailed significance at p <0.05 will be used for univariate frequency comparisons. Test for association at each marker will be performed, for example, one SNP at a time. Since this analysis is only powered to generate hypotheses, no correction will be made for multiple hypothesis testing. Variables significant in any of these analyses will be fit into a logistic regression model to identify factors that were collectively predictive of treatment failure.

### 4. Required equipment and supplies

The drugs to be used in the study will be supplied by the Cambodian Ministry of Health. The required equipment for the molecular marker assays, including the PCR, RT-PCR and Luminex instruments are available at the NAMRU-2/NIPH lab in Phnom Penh, at the Institute Pasteur de Cambodge, and, at the National Malaria Center in Phnom Penh. The reagents required for the assays, probes, primers, PCR and RT-PCR reagents are perishable. Quantities adequate to begin the study are on hand or already ordered as of May 2009. Additional perishable supplies will be purchased as required.

### 5. Budget

|  |  | Year 1 | Year 2 | Total |
| --- | --- | --- | --- | --- |
| Travel |  | 6000 | 6000 | 12000 |
| Reagents |  |  |  |  |
|  | Assay DHFR SNPs | 7000 | 7000 | 14000 |
|  | Assay DHPS SNPs | 4950 | 4950 | 9900 |
|  | Assay Pfcrt SNP | 1900 | 1900 | 3800 |
|  | Assay Pfmdr Copy # | 3680 | 3680 | 7360 |
|  | Assay Pfmdr SNPs | 5500 | 5500 | 11000 |
| Consumables |  | 6000 | 6000 | 12000 |
| Communication |  | 2000 | 2000 | 4000 |
| Locally Hired Staff/Contracts |  | 27600 | 28000 | 55600 |
| Total |  | 64630 | 65030 | 129660 |

## C. ORGANIZATION OF RESEARCH EFFORT

### 1. Duties and Responsibilities of Investigators and Medical Monitor

Dr. Lek Dysoley (National Center for Malaria) is the principal investigator and has overall responsibility for the design and conduct of the study.

Dr. William Rogers (NAMRU-2) is the Navy Lead Investigator and is responsible for adherence to all relevant Navy regulations for the conduct of human subjects research. Dr. Rogers also assists in the design, implementation, and analysis of the study.

Dr. Duong Socheat (National Center for Malaria) will advise on study design and will participate in analysis of the results.

Mrs. Sem Rithy and Mr.Phektra Chim, will conduct the RT-PCR, IC_50_, and Luminex assays.

Dr. Sinuon Muth will serve as the medical monitor. Dr. Muth will ensure compliance to the medical ethics guidelines described above and will act as the point-of-contact for subjects with ethical or medical concerns about their participation in the study.

### 2. Multicenter Organizational Plan for IRB Review and Approval

The protocol will undergo scientific review by the NAMRU-2 Scientific Review Board. Ethical review will be performed by the National Ethics Committee of Cambodia and the NAMRU-2 IRB. The Naval Medical Research Center Office of Research Administration and Bureau of Medicine each provide subsequent quality assurance review, but the study may begin once final approvals by the National Ethics Committee of Cambodia and NAMRU-2 have been granted. Continuing review of the study for NAMRU-2 will be conducted by the IRB of the Naval Medical Research Center, Silver Spring Maryland, pending re-establishment of the NAMRU-2 IRB in Phnom Penh.

### 3. Technology Transfer/Capacity Building/Sample Sharing Plan

The conduct of this protocol will strengthen the capacity of Cambodian scientists at all participating institutions to perform assays to detect molecular markers of drug resistance, including real-time PCR and Luminex flow-cytometry based assays. The use of these assays will, ultimately facilitate monitoring the spread of drug resistant strains of *P. falciparum*. All molecular assays will be performed in Cambodia at one of the collaborating institutions. Samples will be shared freely between the collaborating institutions in Cambodia in order to complete the assays included in this study. It is not anticipated that any biosamples will be sent out of Cambodia in the course of this protocol.

## D. RISKS AND BENEFITS FOR SUBJECTS

### 1. Risks

There are three main risks in the study, the risk of treatment failure due to drug resistance, the risk of known side effects of the three standard anti-malarial regimens used, and risks associated with venipuncture and fingerprick blood sampling.

Treatment failure

There is a small, but very real risk of treatment failure with all three of the standard treatment regimens. There has been recent evidence of diminished efficacy of mefloquine-artesunate in some areas of Cambodia (10), but no such evidence from Oddarmeanchey Province. Recent WHO studies in Western Cambodia found efficacies for both Malarone and piperaquine-DHP of >95%.

#### Side effects of standard anti-malarial drugs

The three treatment regimens to be evaluated are either included in current Cambodian Ministry of Health guidelines for treatment of uncomplicated *P. falciparum* malaria (mefloquine-artesunate and piperaquine-DHA) or are recommended for use in drug resistance containment programs in Cambodia by the World Health Organization (Malarone). Their safety profiles are well understood and their adverse effects are mild compared to the risks of untreated *P. falciparum* malaria. Similarly, the adverse event profile of the rescue therapy, quinine-tetracycline, is well understood. Side effects of anti-malarials used for treatment of acute malaria, rather than for prophylaxis, may be difficult to distinguish from the symptoms of malaria. Possible side effects of the treatment regimens are as follows:

*Mefloquine-artesunate*

Side effects occurring in more than 1% of treated subjects may include dizziness, myalgias, fever, vomiting, headache, chills diarrhea, skin rash, abdominal pain, fatigue, loss of appetite, and tinnitus.

*Piperaquine-DHA*

Side effects occurring in more than 1% of treated subjects may include nausea, vomiting, and anorexia.

*Malarone (atovaquone-proguanil)*

Side effects occurring in more than 1% of treated subjects may include abdominal pain, nausea, vomiting, headache, diarrhea, weakness, loss of appetite and dizziness.

Quinine-doxycycline

Side effects occurring in more than 1% of treated subjects include headache, tinnitus, nausea, vomiting, blurred vision, and vertigo. Cardiac side effects from quinine are a significant risk for intravenous administration, but are very rare following oral administration.

#### Side effects of blood sampling

Venipuncture and finger prick blood sampling carry a risk of mild discomfort and a very small risk of infection. The risk of infection will be minimized by the use of aseptic technique and disposable, sterile needles and lancets.

#### Social or economic risks

Neither malaria infection nor anemia is associated with any social stigma, and there is no local health insurance scheme which could use information about anemia or malaria infection to the disadvantage of any subject. There therefore do not appear to be significant social or economic risks to participating in the study.

### 2. Benefits

Participants in the study will receive one of three standard anti-malarial therapies which make up part of Cambodia’s malaria control program. In addition, they will receive more extensive post-treatment monitoring to detect treatment failure than they would routinely. They will receive prompt rescue therapy in the event of treatment failure. In addition they may receive some satisfaction from contributing to efforts to control malaria in Cambodia.

### 3. Confidentiality and Sample Storage

Case report forms will be stored in locked file cabinets at the field sites and then transported to the National Center for Malaria, where they will be stored in a locked office and will only be accessed by members of the study team or officials with responsibility for study oversight.

Samples collected as part of this study will be maintained in locked drawers or freezers in the National Malaria Center, the Pasteur Institute, or the NAMRU-2/NIPH laboratory. Samples will be used only for the purposes of the study described in this protocol and any excess will be destroyed upon completion of the study.

### 4. Special Risks to Pregnant or Potentially Pregnant Women Volunteers

Female subjects over age 12 must have a negative urine pregnancy test before enrolling in the study. Pregnant patients will not be enrolled in the study.

### 5. Safety Precautions and Emergency Procedures

The risk of morbidity or mortality resulting from treatment failure will be greatly reduced by the careful monitoring for early detection and treatment of recurrent parasitemia. The side effects of treatment with all of the standard anti-malarial regimens used in this study are well understood. Individuals who have contraindications for or known hypersensitivity to any of the anti-malarials in the study will be excluded from the study. The anti-malarials are well tolerated and the risks are reasonable in treatment of a potentially fatal disease. Risks associated with venipuncture and finger prick blood sampling will be minimized as described above.

### 6. Assessment of Sufficiency of Plans to Deal with Untoward Events or Injuries

This study will be conducted at government health center in a rural area in Cambodia. The research team will keep one physician on site 24 hours a day, 7 days a week for the duration of the study. The supervising physician will always maintain a field medical bag available for emergency care of study subjects. In the event of medical emergencies unrelated to study participation, the study team will facilitate access to the healthcare system to the extent practical.

### 7. Qualification of Medical Monitor and Medical Support Personnel

The Medical Monitor, Dr. Sinuon Muth, is a senior physician at the National Malaria Center with over 20 years experience treating patients with malaria. Dr. Lek Dysoley, the principal investigator, is a physician at the National Malaria Center, with extensive experience in malaria treatment.

## E. DESCRIPTION OF THE SYSTEM FOR MAINTENANCE OF RECORDS

### 1. Experimental Data

Experimental data will be maintained in the form of case report forms stored in a locked file cabinet in a locked room in the National Malaria Center; laboratory results will be stored on password protected computers at the National Malaria Center, the Institute Pasteur de Cambodge, and the NAMRU-2/NIPH laboratory.

### 2. Regulatory File

The original research protocol, consent forms, and related documents for protection of human research volunteers will be stored at the National Malaria Center with copies stored NAMRU-2/NIPH. At both institutions these documents will be stored in locked cabinets in locked offices.

### 3. Individual Medical Records

Clinical and laboratory findings from each subject will be kept in individual folders of CRF. These CRFs contains the subject’s name and study code number. This represents the only link established between the subject and the study code number. During the study, the CRFs are held in a secured cabinet with access restricted to research team members with a need to use the CRF. Team members with access to CRFs are counseled on the importance of confidentiality of subject medical records. All NAMRU-2 staff involved in this study have completed Navy Office of Research Administration-sponsored ethics training and hold an IRB certification number. In all electronic records for data analysis, the subject is not referenced by name, but only study code number. This method is designed to protect the privacy of study subject medical information. All CRFs will be held at CNM for at least 5 years in secured cabinets with restricted access.

A report summarizing the findings of this trial will be forwarded to all collaborating institutions (via the co-investigators on this protocol). A record of this research published in a peer-reviewed journal is anticipated.

# Figure 1. Malaria Containment Zones.


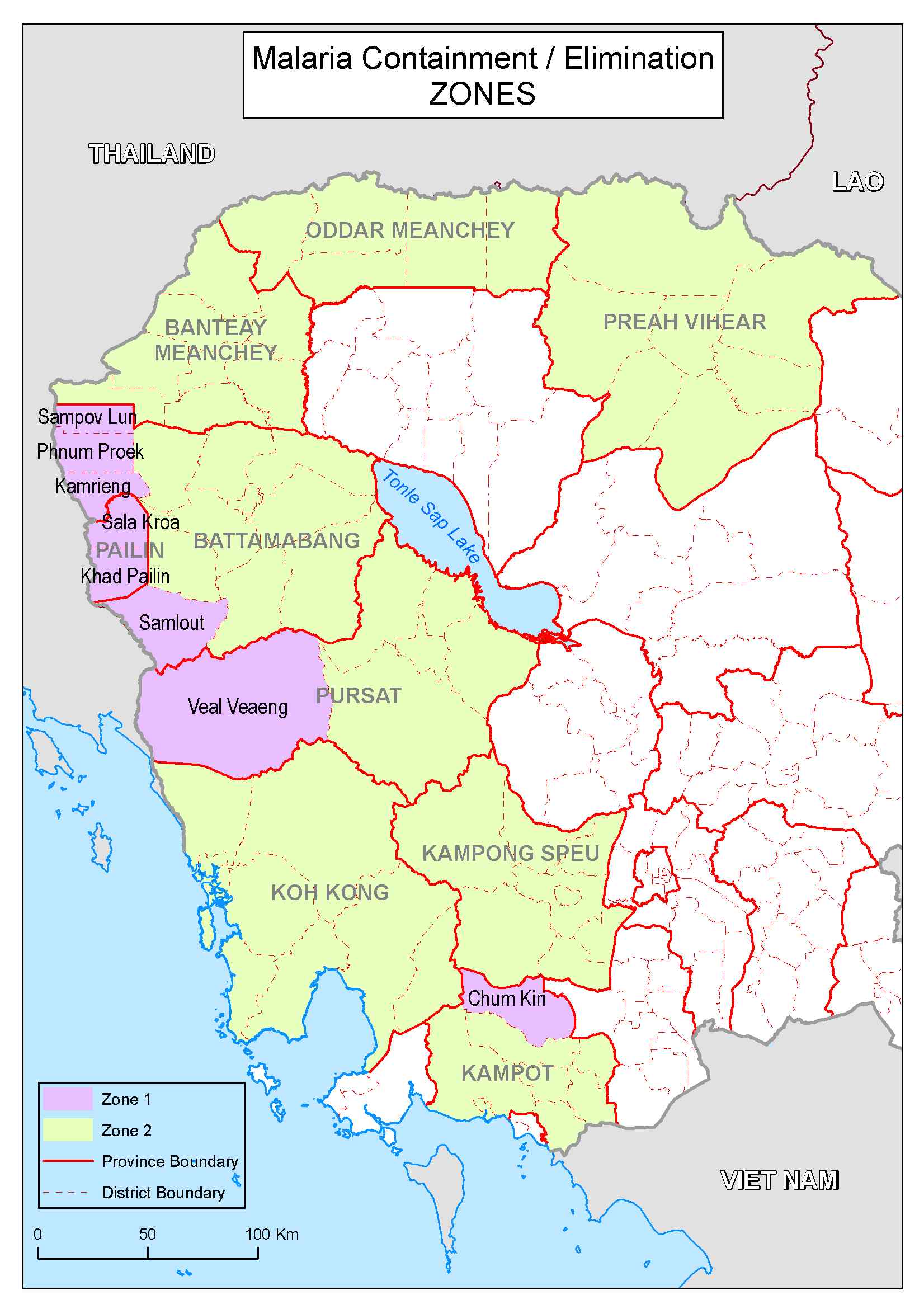


# Appendix A. Case Report Form

1

| Subject number: FTP __ __ __ | Visit Date:  ___ ___/ ___ ___ ___/2009  DD MMM YYYY | **Pre-treatment** |
| --- | --- | --- |

| **SENTINEL SITE** | | | |
| --- | --- | --- | --- |
| Name of the health center: | Locality: | | District: |
| Address: | | Province: | |

| **DEMOGRAPHIC DATA** |
| --- |
| Age: ___ ___ years Gender (please tick 🗹 only one box): ⬜ Male ⬜ Female  Weight: ___ ___ ___ . ___ kg |
|  |

| **PRE-TREATMENT TEMPERATURE** |
| --- |
| Axillary Temperature: ___ ___ . ___ °C  History of fever within previous 24 hours ⬜ Yes ⬜ No |

| **THICK AND THIN BLOOD SMEARS** |
| --- |
| Which species? *P. falciparum* ⬜ *P. vivax** ⬜ *P. ovale** ⬜ *P. malariae** ⬜ Mixed species*  ***NOT ELIGIBLE** |

| **PARASITE DENSITY** |
| --- |
| For *P. falciparum* ***o*nly** (I,000-100,000/µL): >40/200 WBC AND <25/1000 RBC ⬜ Yes ⬜ No*  ***NOT ELIGIBLE** |

| **URINE PREGNANCY TEST** (This section applies only to female patients.) |
| --- |
| Result of pregnancy test: ⬜ Negative ⬜ Positive* **(NOT ELIGIBLE)** |

| **INCLUSION CRITERIA** |
| --- |
| 1. Male or female patients ≥5 year of age 2. Presence of acute symptomatic uncomplicated *P. falciparum* malaria with a diagnosis confirmed by a positive blood smear with *P. falciparum* only (i.e. no mixed infection)    - acceptable range is between 1,000 and 100,000 asexual parasites/µl of blood 3. Measured axillary temperature of ≥37.5°C, tympanic temperature ≥38.0^o^C or history of fever within previous 24 hours 4. Ability to swallow oral medication 5. Negative urine pregnancy test (if female ≥12 years of age) 6. Willingness and ability to comply with study protocol and with study visit schedule for the duration of the study 7. Willingness to give informed consent |
| Does the patient meet all of the inclusion criteria? ⬜ Yes ⬜ No |

| **EXCLUSION CRITERIA** |
| --- |
| 1. Age < 5 years  2. Mixed species infection (e.g. *P. falciparum* and *P. vivax*)  3. Positive urine pregnancy test or lactating  4. History of epilepsy or psychiatric illness  5. Any of the following WHO criteria for severe malaria  Clinical malaria with any one or more of the following clinical manifestations:  i) Unrousable coma or Blantyre Coma Score of 3 or less; coma persists for more than 30 minutes after fits have ceased.  ii) Generalised convulsions lasting more than 30 minutes or more than 2 fits in 24 hours despite cooling  iii) Haemoglobin < 5g/dL  iv) Presence of any of the following; alar flaring, chest recession (intercostals or sub costal), use of accessory muscles of respiration; abnormally deep (acidotic) breathing.  v) Hypoglycaemia (blood glucose < 2.2mM/L[<40 mg/dL])  vi) Circulatory collapse (systolic bp <50mm Hg)  vii) Renal failure (urine output less than 12ml/kg/24hrs; or serum creatinine conc >3.0mg/dL)  viii) Malarial haemoglobinuria  ix) hyperparasitaemia (>100,000 /µl)  x) Jaundice (serum bilirubin ≥ 3.0mg/dL),  xi) Impaired consciousness (but rousable) OR  General danger signs of severe illness in children   - Inability to drink or breastfeed - Repeated vomiting - Recent history of convulsions - Lethargy or unconsciousness - Inability to sit or stand up   6. Serious co-morbid conditions requiring hospitalization (including, but not limited to severe renal or liver disease, uncontrolled diabetes, systemic bacterial infections).  7. On-going antibiotic therapy  8. Known history of hypersensitivity, allergic or adverse reactions to the drugs used in this study  9. Plans to leave area during next 42 days or to be unavailable for scheduled follow-up  10. Nursing mother  11. Any other condition which, in the judgment of the study physician would make participation in the study unsafe for the potential volunteer. |
| Does the patient meet any of the exclusion criteria? ⬜ Yes ⬜ No |
| If yes, please specify reason of exclusion: |

| **PATIENT INFORMED CONSENT AND ASSENT** | | |
| --- | --- | --- |
| Consent Form signed: ⬜ Yes ⬜ No  Assent Form signed: ⬜ Yes ⬜ No | Date: ___ ___/ ___ ___ ___/2009 | Subject Number:  FTP __ __ __ |

| Subject number: FTP __ __ __ | **Treatment**  **(Day 0)** |
| --- | --- |

| **DEMOGRAPHIC DATA** |
| --- |
| Occupation: ____________________________________________________  Possible place of Infection (where did you go in the last 2 weeks?): _______________________________________________ |
| How many days have you been sick? **___ ___** days  History of drug allergy: ⬜ No ⬜ Yes, what drug? __________________________________________________ |

| **HEMATOCRIT** |
| --- |
| Hematocrit ___ ___ % |

| **BLOOD SMEARS FOR QUANTITATIVE PARASITE COUNTS AND QUALITATIVE GAMETOCYTE COUNTS** | | | |
| --- | --- | --- | --- |
| ***P. falciparum*** | | | |
| Was species other than *P. falciparum* detected upon review of slides?  If yes, the patient is to be excluded. | Number of asexual *P. falciparum* parasites | | Presence of *P. falciparum* gametocytes?  ⬜ No ⬜ Yes, rare  ⬜ Yes, easy to find |
|  | /200 WBC | /1,000 RBC  (if>100/HPF on thick) |  |
| ⬜ Yes ⬜ No | ­­­­­­­­­­­­­­­­­___ ___ ___ ___ | ___ ___ ___ |  |

| **PRIOR MEDICATION** | | | | | |
| --- | --- | --- | --- | --- | --- |
| Has the patient taken any prior antimalarial medication? ⬜ Yes ⬜ No  If yes, please specify on this page. | | | | | |
| * All prior medication, including natural remedies and homeopathic drugs, taken within the previous 14 days should be reported in this section. Either Stop date or Ongoing should be filled in. | | | | | |
| Drug name  *(Generic name)* | Start date/Stop date  (DD/MMM/YYY) | Ongoing  (🗹 if Yes) | Total daily  Dose & unit | Route of administration | Indication for use |
|  | ___ ___/___ ___ ___/2006  ___ ___/___ ___ ___/2006 | ⬜ |  |  |  |
|  | ___ ___/___ ___ ___/2006  ___ ___/___ ___ ___/2006 | ⬜ |  |  |  |

| Subject number:  FTP __ __ __ | Visit Date:  ___ ___/ ___ ___ ___/___ ___ | **Treatment**  **(Day 0)** |
| --- | --- | --- |

| **Physical Examination** |
| --- |
| Time:  ___ ___ : ___ ___ (HH:MM) |
| **Vital Signs** |
| Time of measurement: ___ ___ : ___ ___ (HH:MM) |
| Systolic blood pressure (SBP)/Diastolic blood pressure (DBP): ___ ___ ___ / ___ ___ ___ |
| Heart Rate: ___ ___ ___ b.p.m. |
| Axillary Temperature: ___ ___ . ___ °C |

|  | Please tick 🗹 the applicable box | | |  |
| --- | --- | --- | --- | --- |
| **Body System** | **Normal** | **Abnormal** | **Not examined** | **Findings (if abnormal)** |
| General Appearance | ⬜ | ⬜ | ⬜ |  |
| Head and Eyes | ⬜ | ⬜ | ⬜ |  |
| Ears, Nose and Throat | ⬜ | ⬜ | ⬜ |  |
| Chest and Lungs | ⬜ | ⬜ | ⬜ |  |
| Cardiovascular | ⬜ | ⬜ | ⬜ |  |
| Abdomen | ⬜ | ⬜ | ⬜ |  |
| Neurological | ⬜ | ⬜ | ⬜ |  |
| Lymphatic (excluding head and neck) | ⬜ | ⬜ | ⬜ |  |
| Musculo-skeleton | ⬜ | ⬜ | ⬜ |  |
| Other, specify: | ⬜ | ⬜ | ⬜ |  |
| Other, specify: | ⬜ | ⬜ | ⬜ |  |
| Other, specify | ⬜ | ⬜ | ⬜ |  |
| **Subject number:** FTP ______ | | | | |

| **Assessment of Clinical signs and Symptoms** | | | |
| --- | --- | --- | --- |
| **Signs and Symptoms** | **Present** | **Absent** | **Note** |
| Rigors/Chills | ⬜ | ⬜ |  |
| Sweating | ⬜ | ⬜ |  |
| Headache | ⬜ | ⬜ |  |
| Cough | ⬜ | ⬜ |  |
| Nausea | ⬜ | ⬜ |  |
| Vomiting | ⬜ | ⬜ |  |
| Loss of appetite | ⬜ | ⬜ |  |
| Fatigue | ⬜ | ⬜ |  |
| Myalgia | ⬜ | ⬜ |  |
| Jaundice | ⬜ | ⬜ |  |
| Hepatomegaly | ⬜ | ⬜ |  |
| Splenomegaly | ⬜ | ⬜ |  |
| Other (1) | ⬜ | ⬜ |  |
| Other (2) | ⬜ | ⬜ |  |

| The Examining Physician:  Name Signature |
| --- |

| **STUDY MEDICATION ADMINISTRATION** | | | | |
| --- | --- | --- | --- | --- |
| Name of the **antimalarial** drugs | Time of dosing  (HH:MM) | Number of  tablets | Did the patient  vomit? | Time of vomiting  (HH:MM) |
|  | ___ ___:___ ___ | ___ ___ | ⬜ Yes ⬜ No | ___ ___:___ ___ |
|  | ___ ___:___ ___ | ___ ___ | ⬜ Yes ⬜ No | ___ ___:___ ___ |
|  | ___ ___:___ ___ | ___ ___ | ⬜ Yes ⬜ No | ___ ___:___ ___ |

| **ADJUNCTIVE MEDICATIONS** | | | | |
| --- | --- | --- | --- | --- |
| Name of other medicines | Time of dosing  (HH:MM) | Number of tablets | Did the patient  vomit? | Time of vomiting  (HH:MM) |
|  | ___ ___:___ ___ | ___ ___ | ⬜ Yes ⬜ No | ___ ___:___ ___ |
|  | ___ ___:___ ___ | ___ ___ | ⬜ Yes ⬜ No | ___ ___:___ ___ |

| Subject number: FTP __ __ __ | Date:  ___ ___/ ___ ___ ___/2006  DD MMM YYYY | **Treatment**  **(Day 1)** |
| --- | --- | --- |

| **CLINICAL STATUS** |
| --- |
| Presence of signs of severe or complicated malaria? ⬜ Yes ⬜ No |

| **TEMPERATURE** |
| --- |
| Axillary Temperature: ___ ___ . ___ °C |

| **BLOOD SMEARS FOR QUANTITATIVE PARASITE COUNTS AND QUALITATIVE GAMETOCYTE COUNTS** | | | |
| --- | --- | --- | --- |
| ***P. falciparum*** | | | |
| Is species other than *P. falciparum* present? | Number of  *P. falciparum* parasites (/200 WBC) | | Presence of  *P. falciparum* gametocytes? |
| ⬜ Yes ⬜ No | ___ ___ ___ ___ | | ⬜ Yes ⬜ No |
| **ADVERSE EVENTS** | | | |
| Presence of an adverse event? ⬜ Yes ⬜ No | | Is it a serious adverse event? ⬜ Yes ⬜ No  If yes, contact Dr. Lek Dysoley (Tel. 855-23-211-926) within 24 hours | |
| Nature of the adverse event: | | | |

| **STUDY MEDICATION ADMINISTRATION** | | | | |
| --- | --- | --- | --- | --- |
| Name of the **antimalarial** drugs | Time of dosing  (HH:MM) | Number of  tablets | Did the patient  vomit? | Time of vomiting  (HH:MM) |
|  | ___ ___:___ ___ | ___ ___ | ⬜ Yes ⬜ No | ___ ___:___ ___ |
|  | ___ ___:___ ___ | ___ ___ | ⬜ Yes ⬜ No | ___ ___:___ ___ |
|  | ___ ___:___ ___ | ___ ___ | ⬜ Yes ⬜ No | ___ ___:___ ___ |

| **ADJUNCTIVE MEDICATION** | | | | |
| --- | --- | --- | --- | --- |
| Name of other medicines | Time of dosing  (HH:MM) | Number of tablets | Did the patient  vomit? | Time of vomiting  (HH:MM) |
|  | ___ ___:___ ___ | ___ ___ | ⬜ Yes ⬜ No | ___ ___:___ ___ |
|  | ___ ___:___ ___ | ___ ___ | ⬜ Yes ⬜ No | ___ ___:___ ___ |

| Subject number: FTP __ __ __ | Date:  ___ ___/ ___ ___ ___/2009  DD MMM YYYY | **Treatment**  **(Day 2)** |
| --- | --- | --- |

| **CLINICAL STATUS** |
| --- |
| Presence of signs of severe or complicated malaria? ⬜ Yes ⬜ No |

| **TEMPERATURE** |
| --- |
| Axillary Temperature: ___ ___ . ___ °C |

| **BLOOD SMEARS FOR QUANTITATIVE PARASITE COUNTS AND QUALITATIVE GAMETOCYTE COUNTS** | | | |
| --- | --- | --- | --- |
| ***P. falciparum*** | | | |
| Is species other than *P. falciparum* present? | Number of  *P. falciparum* parasites (/200 WBC) | | Presence of  *P. falciparum* gametocytes? |
| ⬜ Yes ⬜ No | ___ ___ ___ ___ | | ⬜ Yes ⬜ No |
| **ADVERSE EVENTS** | | | |
| Presence of an adverse event? ⬜ Yes ⬜ No | | Is it a serious adverse event? ⬜ Yes ⬜ No  If yes, contact Dr. Lek Dysoley (Tel. 855-23-211-926) within 24 hours | |
| Nature of the adverse event: | | | |

| **STUDY MEDICATION ADMINISTRATION** | | | | |
| --- | --- | --- | --- | --- |
| Name of the **antimalarial** drugs | Time of dosing  (HH:MM) | Number of  tablets | Did the patient  vomit? | Time of vomiting  (HH:MM) |
|  | ___ ___:___ ___ | ___ ___ | ⬜ Yes ⬜ No | ___ ___:___ ___ |
|  | ___ ___:___ ___ | ___ ___ | ⬜ Yes ⬜ No | ___ ___:___ ___ |
|  | ___ ___:___ ___ | ___ ___ | ⬜ Yes ⬜ No | ___ ___:___ ___ |

| **ADJUNCTIVE MEDICATION** | | | | |
| --- | --- | --- | --- | --- |
| Name of other medicines | Time of dosing  (HH:MM) | Number of tablets | Did the patient  vomit? | Time of vomiting  (HH:MM) |
|  | ___ ___:___ ___ | ___ ___ | ⬜ Yes ⬜ No | ___ ___:___ ___ |
|  | ___ ___:___ ___ | ___ ___ | ⬜ Yes ⬜ No | ___ ___:___ ___ |

| Subject number: FTP __ __ __ | Date:  ___ ___/ ___ ___ ___/2009  DD MMM YYYY | **Treatment**  **(Day 3)** |
| --- | --- | --- |

| **CLINICAL STATUS** |
| --- |
| Presence of signs of severe or complicated malaria? ⬜ Yes ⬜ No |

| **TEMPERATURE** |
| --- |
| Axillary Temperature: ___ ___ . ___ °C |

| **BLOOD SMEARS FOR QUANTITATIVE PARASITE COUNTS AND QUALITATIVE GAMETOCYTE COUNTS** | | | |
| --- | --- | --- | --- |
| ***P. falciparum*** | | | |
| Is species other than *P. falciparum* present? | Number of  *P. falciparum* parasites (/200 WBC) | | Presence of  *P. falciparum* gametocytes? |
| ⬜ Yes ⬜ No | ___ ___ ___ ___ | | ⬜ Yes ⬜ No |
| **ADVERSE EVENTS** | | | |
| Presence of an adverse event? ⬜ Yes ⬜ No | | Is it a serious adverse event? ⬜ Yes ⬜ No  If yes, contact Dr. Lek Dysoley (Tel. 855-23-211-926) within 24 hours | |
| Nature of the adverse event: | | | |

| **STUDY MEDICATION ADMINISTRATION** | | | | |
| --- | --- | --- | --- | --- |
| Name of the **antimalarial** drugs | Time of dosing  (HH:MM) | Number of  tablets | Did the patient  vomit? | Time of vomiting  (HH:MM) |
|  | ___ ___:___ ___ | ___ ___ | ⬜ Yes ⬜ No | ___ ___:___ ___ |
|  | ___ ___:___ ___ | ___ ___ | ⬜ Yes ⬜ No | ___ ___:___ ___ |
|  | ___ ___:___ ___ | ___ ___ | ⬜ Yes ⬜ No | ___ ___:___ ___ |

| **ADJUNCTIVE MEDICATION** | | | | |
| --- | --- | --- | --- | --- |
| Name of other medicines | Time of dosing  (HH:MM) | Number of tablets | Did the patient  vomit? | Time of vomiting  (HH:MM) |
|  | ___ ___:___ ___ | ___ ___ | ⬜ Yes ⬜ No | ___ ___:___ ___ |
|  | ___ ___:___ ___ | ___ ___ | ⬜ Yes ⬜ No | ___ ___:___ ___ |

| Subject number: FTP __ __ __ | Date:  ___ ___/ ___ ___ ___/2009  DD MMM YYYY | **Treatment**  **(Day 7)** |
| --- | --- | --- |

| **CLINICAL STATUS** |
| --- |
| Presence of signs of severe or complicated malaria? ⬜ Yes ⬜ No |

| **TEMPERATURE** |
| --- |
| Axillary Temperature: ___ ___ . ___ °C |

| **BLOOD SMEARS FOR QUANTITATIVE PARASITE COUNTS AND QUALITATIVE GAMETOCYTE COUNTS** | | | |
| --- | --- | --- | --- |
| ***P. falciparum*** | | | |
| Is species other than *P. falciparum* present? | Number of  *P. falciparum* parasites (/200 WBC) | | Presence of  *P. falciparum* gametocytes? |
| ⬜ Yes ⬜ No | ___ ___ ___ ___ | | ⬜ Yes ⬜ No |
| **ADVERSE EVENTS** | | | |
| Presence of an adverse event? ⬜ Yes ⬜ No | | Is it a serious adverse event? ⬜ Yes ⬜ No  If yes, contact Dr. Lek Dysoley (Tel. 855-23-211-926) within 24 hours | |
| Nature of the adverse event: | | | |

| **STUDY MEDICATION ADMINISTRATION** | | | | |
| --- | --- | --- | --- | --- |
| Name of the **antimalarial** drugs | Time of dosing  (HH:MM) | Number of  tablets | Did the patient  vomit? | Time of vomiting  (HH:MM) |
|  | ___ ___:___ ___ | ___ ___ | ⬜ Yes ⬜ No | ___ ___:___ ___ |
|  | ___ ___:___ ___ | ___ ___ | ⬜ Yes ⬜ No | ___ ___:___ ___ |
|  | ___ ___:___ ___ | ___ ___ | ⬜ Yes ⬜ No | ___ ___:___ ___ |

| **ADJUNCTIVE MEDICATION** | | | | |
| --- | --- | --- | --- | --- |
| Name of other medicines | Time of dosing  (HH:MM) | Number of tablets | Did the patient  vomit? | Time of vomiting  (HH:MM) |
|  | ___ ___:___ ___ | ___ ___ | ⬜ Yes ⬜ No | ___ ___:___ ___ |
|  | ___ ___:___ ___ | ___ ___ | ⬜ Yes ⬜ No | ___ ___:___ ___ |

| Subject number: FTP __ __ __ | Date:  ___ ___/ ___ ___ ___/2009  DD MMM YYYY | **Treatment**  **(Day __)**  14,21,28,35,X |
| --- | --- | --- |

| **CLINICAL STATUS** |
| --- |
| Presence of signs of severe or complicated malaria? ⬜ Yes ⬜ No |

| **TEMPERATURE** |
| --- |
| Axillary Temperature: ___ ___ . ___ °C |

| **BLOOD SMEARS FOR QUANTITATIVE PARASITE COUNTS AND QUALITATIVE GAMETOCYTE COUNTS** | | | |
| --- | --- | --- | --- |
| ***P. falciparum*** | | | |
| Is species other than *P. falciparum* present? | Number of  *P. falciparum* parasites (/200 WBC) | | Presence of  *P. falciparum* gametocytes? |
| ⬜ Yes ⬜ No | ___ ___ ___ ___ | | ⬜ Yes ⬜ No |
| **ADVERSE EVENTS** | | | |
| Presence of an adverse event? ⬜ Yes ⬜ No | | Is it a serious adverse event? ⬜ Yes ⬜ No  If yes, contact Dr. Lek Dysoley (Tel. 855-23-211-926) within 24 hours | |
| Nature of the adverse event: | | | |

| **STUDY MEDICATION ADMINISTRATION** | | | | |
| --- | --- | --- | --- | --- |
| Name of the **antimalarial** drugs | Time of dosing  (HH:MM) | Number of  tablets | Did the patient  vomit? | Time of vomiting  (HH:MM) |
|  | ___ ___:___ ___ | ___ ___ | ⬜ Yes ⬜ No | ___ ___:___ ___ |
|  | ___ ___:___ ___ | ___ ___ | ⬜ Yes ⬜ No | ___ ___:___ ___ |
|  | ___ ___:___ ___ | ___ ___ | ⬜ Yes ⬜ No | ___ ___:___ ___ |

| **ADJUNCTIVE MEDICATION** | | | | |
| --- | --- | --- | --- | --- |
| Name of other medicines | Time of dosing  (HH:MM) | Number of tablets | Did the patient  vomit? | Time of vomiting  (HH:MM) |
|  | ___ ___:___ ___ | ___ ___ | ⬜ Yes ⬜ No | ___ ___:___ ___ |
|  | ___ ___:___ ___ | ___ ___ | ⬜ Yes ⬜ No | ___ ___:___ ___ |

| Subject number: FTP __ __ __ | Date:  ___ ___/ ___ ___ ___/2009  DD MMM YYYY | **Treatment**  **(Day 42**) |
| --- | --- | --- |

| **CLINICAL STATUS** |
| --- |
| Presence of signs of severe or complicated malaria? ⬜ Yes ⬜ No |

| **TEMPERATURE** |
| --- |
| Axillary Temperature: ___ ___ . ___ °C |

| **BLOOD SMEARS FOR QUANTITATIVE PARASITE COUNTS AND QUALITATIVE GAMETOCYTE COUNTS** | | |
| --- | --- | --- |
| ***P. falciparum*** | | |
| Is species other than *P. falciparum* present? | Number of  *P. falciparum* parasites (/200 WBC) | Presence of  *P. falciparum* gametocytes? |
| ⬜ Yes ⬜ No | ___ ___ ___ ___ | ⬜ Yes ⬜ No |

| **ADVERSE EVENTS** | |
| --- | --- |
| Presence of an adverse event? ⬜ Yes ⬜ No | Is it a serious adverse event? ⬜ Yes ⬜ No  If yes, contact Dr. Lek Dysoley (Tel. 855-23-211-926) within 24 hours |
| Nature of the adverse event: | |

| **STUDY MEDICATION ADMINISTRATION** | | | | |
| --- | --- | --- | --- | --- |
| Name of medicines | Time of dosing  (HH:MM) | Number of  tablets | Did the patient  vomit? | Time of vomiting  (HH:MM) |
|  | ___ ___:___ ___ | ___ ___ | ⬜ Yes ⬜ No | ___ ___:___ ___ |
|  | ___ ___:___ ___ | ___ ___ | ⬜ Yes ⬜ No | ___ ___:___ ___ |

| Outcome : ⬜ ACPR ⬜ ETF ⬜ LCF ⬜ LPF ⬜ LFU ⬜ WTH |
| --- |
| Date of Outcome: ___ ___/___ ___ ___/2009 |
| PCR Results: ⬜ Reinfection ⬜ Recrudescence ⬜ Undetermined ⬜ Not available |
| After PCR correction: ⬜ ACPR ⬜ ETF ⬜ LCF ⬜ LPF |
| Reason for withdrawal: |
| Other comments: |

#

# Appendix B

**Volunteer Informed Consent Document**

Efficacy of three standard therapies for uncomplicated *P. falciparum* malaria in Cambodia

**PURPOSE**

You^†^ (or your child) and about 300 other malaria patients in Cambodia have been invited to participate in a project entitled, “Efficacy of three standard therapies for uncomplicated *P. falciparum* malaria in Cambodia.” The National Malaria Center is conducting this study in collaboration with the Institute Pasteur de Cambodge, and the U.S. Naval Medical Research Unit No. 2 (US NAMRU-2). Malaria is a disease caused by a parasite transmitted by mosquitoes that can cause fever, muscle aches, cough, headache, nausea, vomiting, abdominal cramping, diarrhea, seizures, weakness, unconsciousness, and death.

The purpose of this study is to learn whether three different treatments for malaria work well in this part of Cambodia. All three treatments are recommended by the Ministry of Health and the World Health Organization. Mefloquine-artesunate is the standard treatment for malaria in most of Cambodia. Piperaquine-DHA (Artekin) is recommended as standard treatment in some parts of western and southern Cambodia, where mefloquine-artesunate may not work well. Malarone (atovaquone-proguanil) is recommended by the WHO for treatment of malaria in malaria control campaigns in western Cambodia. Knowing how well each of these treatments works will help plan malaria control programs in Cambodia.

**YOUR PARTICIPATION AND RIGHTS**

If you agree to participate in the study the study team will choose one of the three malaria treatments for you by chance (like in a lottery). You will need to take the medications today, and for the next two days. You will take the medication here at the health center; after each dose you will remain at the health center for at least an hour to make sure you do not vomit. If you do vomit within one hour, you will get another dose of the same medication. You may either stay at the health center until you have received the last dose (the day after tomorrow), or, if it is more convenient, you may sleep at home, but return to the health center tomorrow and the next day to take the medication.

We will take small blood samples to learn whether the treatment is working. Today we will use a needle to collect a small sample of blood (one teaspoonful) from your vein. Then, on the next two days, one week from today, and again every week for 6 weeks we will give you a simple physical exam and collect a few drops of blood from your finger to see whether the malaria has been cured. If you feel sick at any time during the next 6 weeks, please come back so that we can check you for malaria.

If we find that your malaria has come back we will offer treatment with a full 7-day course of quinine plus tetracycline (approved by the Cambodian Ministry of Health for the treatment for malaria if not cured by the first-line treatments described above). This treatment is harder to take (longer time and with more side effects), but we know it works very well against malaria.

Your participation in this study is completely voluntary. You can decline participation for any reason. If you do not want to participate, you will still receive free treatment for your malaria. If you agree to participate, you may withdraw at any point without penalty or loss of benefits to which you are otherwise entitled.

**WHAT YOUR BLOOD WILL BE USED FOR**

Your blood sample will be used only for the purpose of this project as stated above. We will test the blood to see whether your malaria has been cured. If there are malaria parasites in the sample, we will test them to see if they might be resistant to anti-malarial drugs. Your blood will not be tested for human genetics, blood type, markers of immune status, or infectious agents such as the AIDS virus or hepatitis virus.

**PROCEDURES**

A well-trained doctor or nurse will draw blood from the vein in your arm today. He will first clean the skin above the vein, and the will use a sterile needle and syringe to collect a small amount of blood, approximately one teaspoon. On the other days that you return to be checked for malaria a trained health care worker will clean the tip of your finger and collect a few drops of blood by pricking your finger with a sterile lancet.

**RISKS AND DISCOMFORTS**

1). Failure of Treatment. There is a small chance that any of the drugs might fail to cure your malaria, leaving you in danger of severe disease. For this reason we will check your blood frequently after treatment to make sure that your malaria has been cured. If we find that your malaria has come back we will offer treatment with a full 7-day course of quinine plus tetracycline (approved by the Cambodian Ministry of Health for the treatment for malaria if not cured by the first-line treatments described above).

2). Side effects from the drugs. As with any drug, a small fraction of people may have side effects and reactions that cannot be predicted. The most common side effects of the drugs you may receive are dizziness, muscle pains, fever, vomiting, headache, chills diarrhea, skin rash, abdominal pain, fatigue, loss of appetite, blurred vision, and ringing in the ears. These side effects generally go away quickly once treatment is finished.

You must always come quickly to see a study doctor if you have any illness, injury, or drug reaction. A medical doctor is available 24 hours a day, seven days a week to see you during the study. If you have a medical problem resulting directly from your participation in the study, we will help you get medical care for it.

3) Special warning for pregnant women. One of the study drugs, Malarone, is not recommended for pregnant women. Therefore, you cannot participate in the study if you are pregnant. To be sure you are not pregnant, we will perform a urine pregnancy test before allowing you to participate. If you are pregnant, we will still provide you with treatment for malaria, but we cannot enroll you in the study.

4) Risks and discomforts from blood sampling.

Taking blood from the finger causes a brief sharp pain. There is a very low risk of infection where the needle is stuck in the finger. However, the research team knows how to minimize that risk and they will help you if a problem occurs. If you become dizzy with pain or the sight of blood, please tell the person taking the blood, so that you can lie down.

Drawing blood from your arm may cause brief discomfort, and there is a slight risk of infection as well as bleeding, or fainting that may require medical attention. Such events are rare. Bruising is more common but usually disappears in a few days. All available precautions will be taken to prevent these. Only an experienced technician or nurse or doctor will draw your blood with sterile, one-time use equipment under aseptic technique.

**BENEFITS**

You will not receive any monetary compensation for participation in this study. Malaria can kill you without effective treatment. If you enroll in this study, the doctors will monitor the treatment and its outcome closely to ensure that you are cured of the infection. Results from this research will help the Ministry of Health design better treatment regimens for malaria for your community in the future. It will allow measures to be taken that may reduce the spread of drug resistant malaria in Cambodia.

**SAFETY AND CONFIDENTIALITY**

All investigators and the Medical Monitor will ensure your safety and confidentiality during and after blood drawing. Your name will not appear on your blood samples. The consent form and information obtained from you will be stored at the National Malaria Center lab in Phnom Penh, in locked, secure offices and file cabinets.

**COMPENSATION**

You will receive no money or other direct compensation for your participation. However, any costs incurred by you for any medical conditions that are a direct result of your blood donation and require medical intervention will be the responsibility of US NAMRU-2. You will suffer no financial loss as a result of participation. You will be provided with food during your stay at the health center/hospital in the next few days and will receive a standard transportation fee for your weekly return to the health center/hospital during follow-up.

**QUESTIONS**

If you have any questions or concern about complications that arise after your blood is taken, you can call or visit a physician/health officer at the location where your blood was drawn and you will be evaluated/ or referred for evaluation in a timely manner. Alternatively, you may contact Dr. Lek Dysoley of the National Malaria Center (CNM), Ministry of Health, Phnom Penh (Tel. 855-23-211-926), or Dr. Sinuon Muth, at the National Malaria Center, Phnom Penh (Tel. 855-23-211-926). If you have questions about your rights as a study subject, you may contact Dr. David Fryauff, the Chairman of the Institutional Review Board at NMRC, Silver Spring, MD (Tel. 301-319-7588). If you do not have phone access, the head of the clinic where your blood was drawn can help you contact them.

Signature: You have certified that you have received a copy of this consent form and understand that signing this form verifies your willingness to voluntarily participate in the study yourself (or on behalf of your child).

NAME:_________________________ AGE:___________(yrs)

____________________________ _________

Volunteer Consent Signature Date

(adults ≥ 18)

(THUMB PRINT)

___________________ ________________ _________

Parent/Guardian Consent Signature Date

Name & Signature

(only for child <18 years*)

(THUMB PRINT)

___________________ ________________ _________

Witness Name Signature Date

(Required in all cases**)

(THUMB PRINT)

____________________________ ________________ __________

Information Provider Name Signature Date

* Children aged less than 5 years old are ineligible to participate

** Cannot be study team member.

^†^*Throughout this information sheet, “you” refers to you if you are an adult (>=18 yrs) or “your child” if you are the parent/guardian of a child (<18 yrs) being asked to participate in this research study.*

Reference List

1. **Carnevale, E. P., D. Kouri, J. T. DaRe, D. T. McNamara, I. Mueller, and P. A. Zimmerman**. 2007. A multiplex ligase detection reaction-fluorescent microsphere assay for simultaneous detection of single nucleotide polymorphisms associated with Plasmodium falciparum drug resistance. J.Clin.Microbiol. **45**:752-761.

2. **Denis, M. B., R. Tsuyuoka, Y. Poravuth, T. S. Narann, S. Seila, C. Lim, S. Incardona, P. Lim, R. Sem, D. Socheat, E. M. Christophel, and P. Ringwald**. 2006. Surveillance of the efficacy of artesunate and mefloquine combination for the treatment of uncomplicated falciparum malaria in Cambodia. Trop.Med.Int.Health **11**:1360-1366.

3. **Keen, J., G. A. Farcas, K. Zhong, S. Yohanna, M. W. Dunne, and K. C. Kain**. 2007. Real-time PCR assay for rapid detection and analysis of PfCRT haplotypes of chloroquine-resistant Plasmodium falciparum isolates from India. J.Clin.Microbiol. **45**:2889-2893.

4. **Myint, H. Y., E. A. Ashley, N. P. Day, F. Nosten, and N. J. White**. 2007. Efficacy and safety of dihydroartemisinin-piperaquine. Trans.R.Soc.Trop.Med.Hyg. **101**:858-866.

5. **Na-Bangchang, K. and K. Congpuong**. 2007. Current malaria status and distribution of drug resistance in East and Southeast Asia with special focus to Thailand. Tohoku J.Exp.Med. **211**:99-113.

6. **Nelson, A. L., A. Purfield, P. McDaniel, N. Uthaimongkol, N. Buathong, S. Sriwichai, R. S. Miller, C. Wongsrichanalai, and S. R. Meshnick**. 2005. pfmdr1 genotyping and in vivo mefloquine resistance on the Thai-Myanmar border. Am.J.Trop.Med.Hyg. **72**:586-592.

7. **Pickard, A. L., C. Wongsrichanalai, A. Purfield, D. Kamwendo, K. Emery, C. Zalewski, F. Kawamoto, R. S. Miller, and S. R. Meshnick**. 2003. Resistance to antimalarials in Southeast Asia and genetic polymorphisms in pfmdr1. Antimicrob.Agents Chemother. **47**:2418-2423.

8. **Price, R. N., G. Dorsey, E. A. Ashley, K. I. Barnes, J. K. Baird, U. D'Alessandro, P. J. Guerin, M. K. Laufer, I. Naidoo, F. Nosten, P. Olliaro, C. V. Plowe, P. Ringwald, C. H. Sibley, K. Stepniewska, and N. J. White**. 2007. World Antimalarial Resistance Network I: clinical efficacy of antimalarial drugs. Malar.J. **6**:119.

9. **Purfield, A., A. Nelson, A. Laoboonchai, K. Congpuong, P. McDaniel, R. S. Miller, K. Welch, C. Wongsrichanalai, and S. R. Meshnick**. 2004. A new method for detection of pfmdr1 mutations in Plasmodium falciparum DNA using real-time PCR. Malar.J. **3**:9.

10. **Rogers, W. O., R. Sem, T. Thong, C. Phektra, P. Lim, S. Muth, S. Duong, F. Ariey, and C. Wongsrichanalai**. 2009. Failure of Artesunate-Mefloquine Combination Therapy for Uncomplicated *P. Falciparum* Malaria in southern Cambodia. Malaria J. **8**:10-18.

11. **Temu, E. A., I. Kimani, N. Tuno, H. Kawada, J. N. Minjas, and M. Takagi**. 2006. Monitoring chloroquine resistance using Plasmodium falciparum parasites isolated from wild mosquitoes in Tanzania. Am.J.Trop.Med.Hyg. **75**:1182-1187.

12. **Trape, J. F.** 2001. The public health impact of chloroquine resistance in Africa. Am.J.Trop.Med.Hyg. **64**:12-17.

13. **Vijaykadga, S., C. Rojanawatsirivej, S. Cholpol, D. Phoungmanee, A. Nakavej, and C. Wongsrichanalai**. 2006. In vivo sensitivity monitoring of mefloquine monotherapy and artesunate-mefloquine combinations for the treatment of uncomplicated falciparum malaria in Thailand in 2003. Trop.Med.Int.Health **11**:211-219.

14. **Wilson, C. M., S. K. Volkman, S. Thaithong, R. K. Martin, D. E. Kyle, W. K. Milhous, and D. F. Wirth**. 1993. Amplification of pfmdr 1 associated with mefloquine and halofantrine resistance in Plasmodium falciparum from Thailand. Mol.Biochem.Parasitol. **57**:151-160.

15. **Wilson, P. E., A. P. Alker, and S. R. Meshnick**. 2005. Real-time PCR methods for monitoring antimalarial drug resistance. Trends Parasitol. **21**:278-283.

16. **World Health Organization**. 2003. Assessment and Monitoring of Antimalarial Drug Efficacy for the Treatment of Uncomplicated Falciparum MalariaWHO, Geneva.

17. **Zucker, J. R., T. K. Ruebush, C. Obonyo, J. Otieno, and C. C. Campbell**. 2003. The mortality consequences of the continued use of chloroquine in Africa: experience in Siaya, western Kenya. Am.J.Trop.Med.Hyg. **68**:386-390.
