# Supplementary material for: Efficacy of three anti-malarial regimens for uncomplicated Plasmodium falciparum malaria in Cambodia, 2009–2011: a randomized controlled trial and brief review
Source: Malar J. 2022 Sep 7;21:259. doi: 10.1186/s12936-022-04279-3 (PMC9450427; doi:10.1186/s12936-022-04279-3)
Supplement: Supplementary file 2 — Additional file 2: Table S1. Pfmdr1 copy numbers for AS/MQ failures. [file 12936_2022_4279_MOESM2_ESM.docx]

**Table S1. *Pfmdr1* copy numbers for AS/MQ failures**

| Subject Number | Day 0 *Pfmdr1* copy number | Day of Recrudescence *Pfmdr1* copy number |
| --- | --- | --- |
| FTP027 | 2.46 | 3.39 |
| FTP033 | 3.52 | 7.81 |
| FTP064 | 5.07 | 3.87 |
| FTP119 | 4.62 | 4.12 |
| FTP133 | 3.62 | 1.71 |
| FTP153 | 3.05 | 3.12 |
| FTP161 | 3.99 | 4.69 |
| FTP168 | 2.58 | 1.25 |
| FTP170 | 3.70 | 7.39 |
| FTP171 | 4.50 | * |
| FTP187 | 2.28 | 3.46 |
| FTP195 | 1.48 | 1.39 |

* Subject FTP171 did not have *Pfmdr1* copy number available on the Day of Recrudescence.
